# Crystal Phase Engineering and Light Emission from 4H-SiGe Nanowires

**DOI:** 10.1021/acsnanoscienceau.6c00062

**Published:** 2026-08-19

**Authors:** Riccardo Farina, Hafssa Ameziane, Victor van Lange, Pedro Borlido, Gilles Patriarche, Charles Renard, Erik P. A. M. Bakkers, Silvana Botti, Laetitia Vincent, Jos E. M. Haverkort

**Affiliations:** † 3169 Eindhoven University of Technology, De Groene Loper 19, Eindhoven 5612 AZ, The Netherlands; ‡ 129826 Université Paris-Saclay, CNRS, Centre de Nanosciences et de Nanotechnologies, Palaiseau F-91120, France; § CFisUC, Department of Physics, University of Coimbra, Rua Larga, Coimbra 3004-516, Portugal; ∥ Research Center Future Energy Materials and Systems, Interdisciplinary Centre for Advanced Materials Simulation, Faculty of Physics and Astronomy, 9378 Ruhr University Bochum, Universitätsstrasse 150, Bochum 44801, Germany

**Keywords:** 4H polytype, hexagonal crystal phase, metastable, Si, Ge, direct bandgap, light emission, nanowires

## Abstract

Silicon germanium (SiGe) semiconductors are driving our modern society based on electronic processing devices. However, the inherent indirect bandgap alignment of SiGe prohibits efficient light emission. Crystal phase engineering of SiGe semiconductors allows for direct bandgap hexagonal 2H, 4H, and 6H polytypes. Here, we report the growth of 4H-Ge and 4H-SiGe nanowires with high crystal phase purity. We exploit crystal structure transfer by epitaxial growth of 4H-SiGe nanowires on a 4H-ZnS substrate. Although theory predicts that 4H-Ge exhibits a parity-forbidden fundamental transition, we report direct bandgap light emission from both 4H-Ge and 4H-SiGe nanowires, as well as tunability of the emission wavelength with composition. We find effective recombination lifetimes of 55 and 400 μs for 4H-Ge and 4H-Si_0.2_Ge_0.8_, respectively. We finally observe excellent agreement between *ab initio* density functional theory and experiment on the bandgap and the radiative lifetimes of the 4H-SiGe alloys.

## Graphical abstract

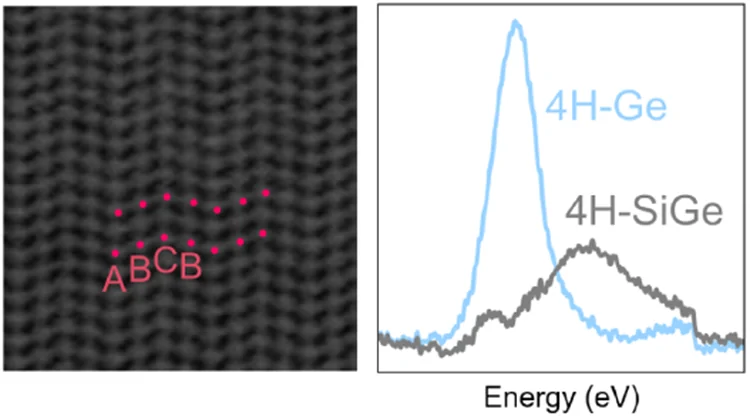

Polytypism in semiconductors^1,2^ provides a powerful route to engineer electronic band structures without altering the chemical composition. By changing the stacking sequence of the closest-packed atomic planes, the crystal symmetry is modified as shown in Figure 1a–d, leading to band folding and symmetry-induced changes in the electronic band structure. In group-IV semiconductors, the transition from the conventional cubic (3C) phase to the purely hexagonal (2H) polytype is a striking example of crystal phase engineering, allowing the transfer of an indirect bandgap 3C-Si_1–*x*_Ge_*x*_ into a direct bandgap 2H-Si_1–*x*_Ge_*x*_ semiconductor for *x* > 0.7. Beyond the 2H polytype, additional hexagonal polytypes^3^ can be formed, such as 4H and 6H-Si_1–*x*_Ge_*x*_. These polytypes combine cubic and hexagonal stacking sequences with hexagonalities of 50% and 33%, respectively. Importantly, theoretical calculations predict^3^ that the 4H-Ge polytype also features a direct bandgap as shown in Figure 1f.

**Figure 1:**
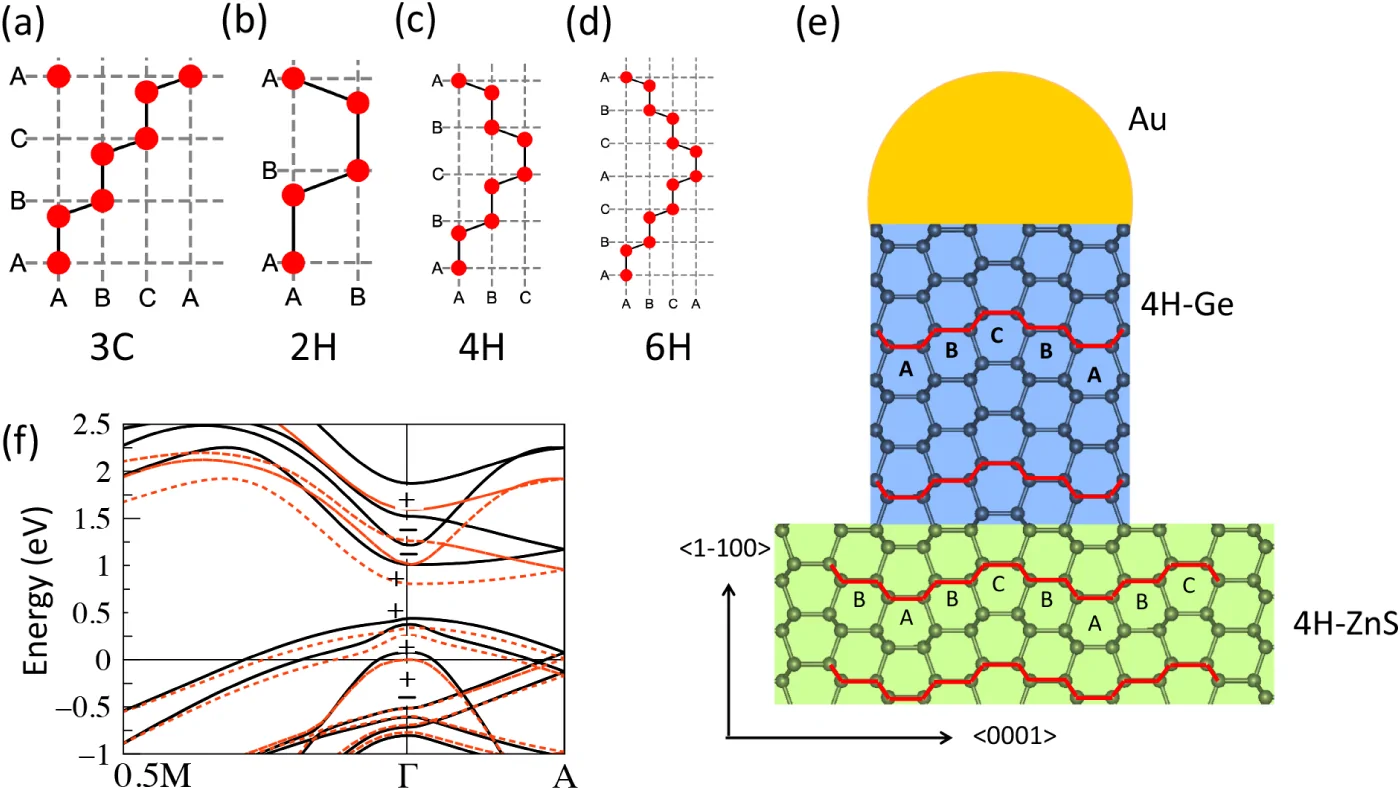
Crystal phase engineering of Ge nanowires showing the atomic stacking sequence of the different Ge polytypes. (a) Cubic 3C-Ge polytype with the characteristic ABC stacking. (b) Hexagonal 2H-Ge polytype showing the characteristic AB stacking. (c) 4H-Ge polytype exhibiting the extended ABCB stacking with 50% hexagonality. (d) 6H-Ge polytype with further extended ABCACB stacking and 33% hexagonality. (e) Schematic illustration for transfer of the 4H stacking sequence toward vertically aligned 4H-Ge nanowires (blue) using a 4H-ZnS substrate (green). (f) Quasiparticle band structure of 4H-Ge showing a direct bandgap. The band structure is calculated with the MBJLDA (red dashed lines) and HSE06 (black lines) functionals and is reproduced with permission from Keller et al.^3^ Copyright 2023 American Physical Society.

The 2H polytype of hexagonal silicon germanium (SiGe) was successfully synthesized by controlled epitaxy of 2H-Si_1–*x*_Ge_*x*_ shells^4,5^ as well as 2H-Ge and 2H-Si nanobranches^6^ on the prismatic side facets of a wurtzite GaAs nanowire (NW) template. The 2H polytype features a direct bandgap, but first-principle density functional theory (DFT)^7–14^ predicts a very small transition matrix element. However, experimentally, 2H-Si_1–*x*_Ge_*x*_ NWs show efficient light emission with a subnanosecond recombination lifetime^1^ and optical gain.^15^ Although pure 2H-Ge does not exhibit an intrinsic absorption band edge in high-resolution scanning transmission electron microscopy electron energy loss spectroscopy (HR-STEM-EELS) measurements,^16^ it also emits bright photoluminescence (PL) with a nanosecond recombination lifetime and high oscillator strength.^17^

The 4H polytype of SiGe is another direct bandgap semiconductor that is chemically compatible with silicon and provides a bandgap energy closer to the 1550 nm window used for telecommunications, making it a potentially promising semiconductor for integrating electronics and photonics into a single chip. Although the formation^18^ of various crystal phases of silicon and germanium has previously been reported by high pressure synthesis^19–21^ nanoindentation^22^ or chemical synthesis^23–25^ the resulting material is often polycrystalline and contains a mixture of various phases and numerous defects. Crystal phase transfer has been shown to accurately replicate hexagonal stacking for epitaxial growth of Au-catalyzed 2H-Ge nanobranches on the {11̅00} m-plane sidewalls of a wurtzite GaAs trunk NW.^6,26^ However, crystal phase transfer beyond the 2H polytype has not yet been reported. It is an open question whether sufficiently pure 4H crystal phase NWs can be realized by growth on m-plane 4H-substrates, as schematically shown in Figure 1e.

The band structure of 4H-Ge has recently been theoretically investigated by Keller et al.^3^ using *ab initio* DFT calculations. Theory predicts that 4H-Ge exhibits a direct bandgap, with a fundamental transition energy in the range of 0.469–0.568 eV, as shown in Figure 1f. Unlike 2H-Ge, which is theoretically predicted to have a very weak but nonzero optical transition matrix element,^8^ 4H-Ge is not expected to emit detectable ground-state PL due to its parity-forbidden lowest-energy optical transition. Currently, it is an open research question whether 4H-Si_1–*x*_Ge_*x*_ semiconductors are capable of efficiently emitting PL from their fundamental direct bandgap transition. Moreover, the question emerges whether the experimental carrier recombination lifetime in 4H-Si_1–*x*_Ge_*x*_ deviates similarly^1,17^ from theory as in 2H-Si_1–*x*_Ge_*x*_. All of these research questions accumulate in the yet unknown prospects of different SiGe polytypes for future optoelectronic device applications.

Here, we report the first experimental realization of pure 4H hexagonal crystal phase Ge and Si_1–*x*_Ge_*x*_ alloys. In this study, these crystals have been synthesized as vertically standing {11̅00}-oriented 4H-Si_1–*x*_Ge_*x*_ NWs grown on a commercially available 4H-ZnS substrate, thus showing robust crystal phase transfer^4,5^ of the 4H crystal phase from the 4H-ZnS substrate to the Ge NWs. The successful growth of high crystal phase purity 4H-Si_1–*x*_Ge_*x*_ NWs is combined with a first study of the fundamental optical properties of this new material. We surprisingly observe clear direct bandgap emission corresponding to the fundamental transition in ensembles of 4H-Ge and 4H-SiGe NWs and show that our experimental observations correspond to the theoretical band structure, the known phonon energies, and the expected transition strengths of 4H-SiGe.

The 4H-Ge NWs have been grown (see Methods) on an epitaxial GaAs layer on top of an m-plane 4H-ZnS substrate, as shown in Figure 2a. The 4H-GaAs layer was required as a crystallographic template because direct growth of Ge NWs on 4H-ZnS did not result in vertically aligned structures, likely due to interface energies that are unfavorable for axial growth. To overcome this limitation, the 4H-GaAs pseudo substrate serves for Au dewetting and subsequent NW growth; see the Supporting Information (SI). High-angle annular dark-field (HAADF) STEM analysis of the 4H-ZnS/4H-GaAs interface (Figure 2c) confirms predominantly hexagonal stacking, although occasional stacking faults are observed, originating from interfacial strain relaxation due to the ≈4.5% in-plane lattice mismatch between GaAs and ZnS.

**Figure 2:**
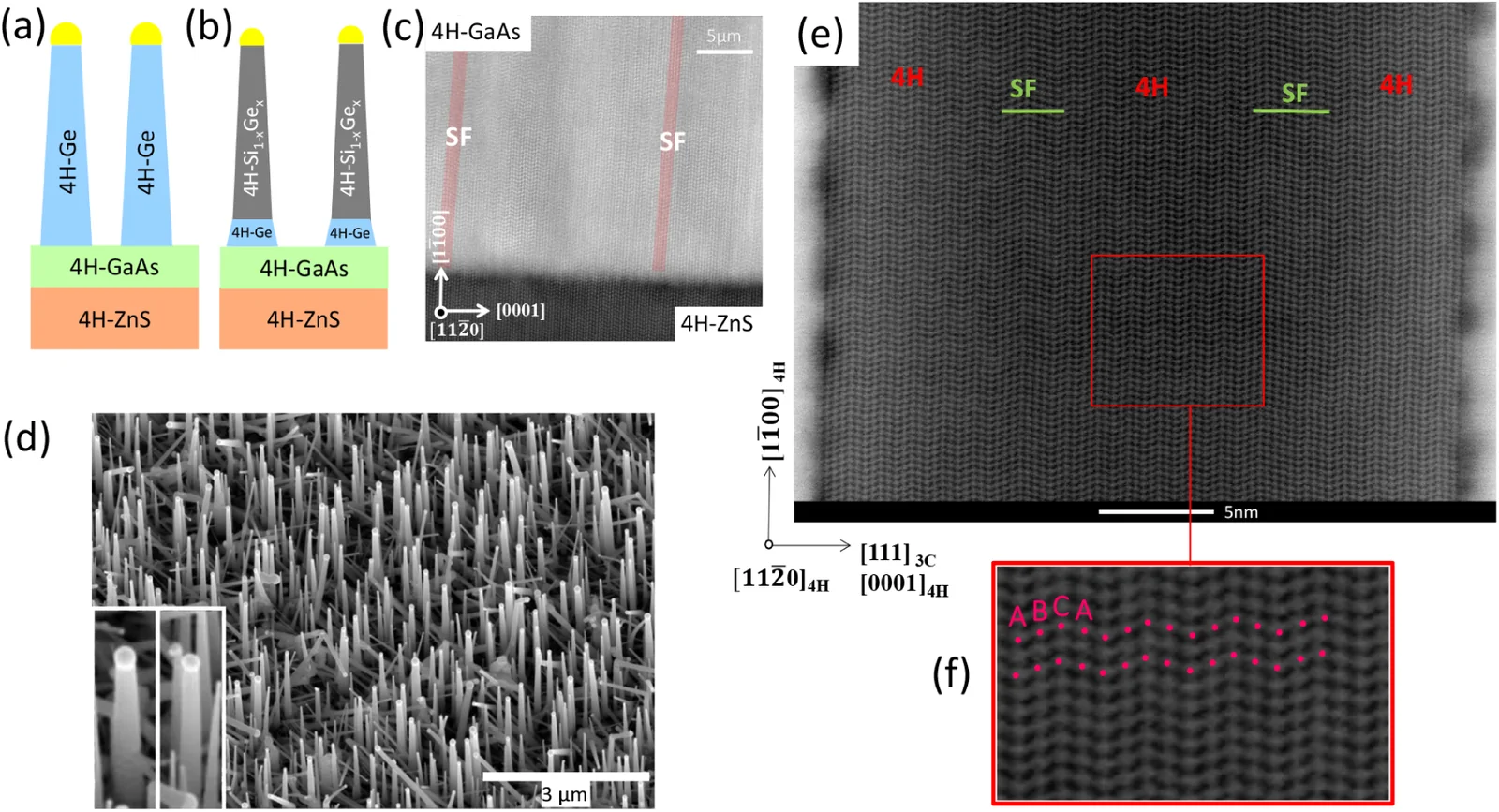
(a) Schematic illustration of vertically aligned 4H-Ge nanowires using a 4H-GaAs/4H-ZnS substrate. (b) Schematic for demonstrating composition control in 4H-Si_1–*x*_Ge*_x_*/Ge nanowire heterostructures. (c) HAADF-STEM micrograph of the 4H-ZnS/4H-GaAs interface acquired along the ⟨11̅20⟩ zone axis, revealing stacking faults inserted within the hexagonal stacking sequence of the 4H-GaAs layer. (d) Tilted-view SEM image showing as-grown vertical Ge nanowires to the GaAs m-plane (11̅00). The inset shows a magnified image of two individual nanowires. (e) Bright field STEM image acquired at the top of the nanowire along the ⟨11̅20⟩ zone axis, showing the 4H crystal structure of a nanowire growing along the ⟨11̅00⟩ direction, with occasional stacking faults. (f) Atomic-resolution bright field STEM image highlighting the 4H stacking sequence (ABCB).

The Ge NWs were subsequently grown using the vapor–liquid–solid (VLS) mechanism employing Au catalyst droplets with an average diameter of approximately 30 nm. The tilted view shown in Figure 2d reveals a high density of well-ordered straight NWs randomly distributed on the pseudo substrate. Most of the NWs are vertical, but some are inclined. The main vertical orientation is confirmed in Figure S1 in the Supporting Information. The bright circular spots correspond to vertical NWs, while linear contrast corresponds to inclined NWs, which have a cubic crystal structure. More details are given in the SI.

The bright field STEM images shown in Figure 2e,f acquired along the ⟨11̅20⟩ zone axis allow unambiguous discrimination between cubic and hexagonal crystal phases.^26^ Importantly, hexagonality is maintained throughout growth and is continuously replicated from the GaAs/Ge interface (Figure S2) to the NW tip (Figure 2e) without introducing I_3_ defects.^27^ However, any defect present in the pseudo substrate propagates along the entire NW.

At the NW base (Figure S2), lateral cubic segments are observed along the sidewalls. This lateral growth develops along the ⟨0001⟩ c-direction, equivalent to ⟨111⟩ in the cubic phase, and is attributed to a vapor–solid (VS) mechanism, competing with axial VLS-growth. The lateral VS growth leads to NW tapering^28,29^ as shown in Figure S3.

Building on the successful stabilization of pure 4H-Ge NWs on the 4H-GaAs template, we demonstrate composition control of 4H crystal phase NWs by growing 4H-Si_1–*x*_Ge_*x*_/Ge NWs (see Methods). To stabilize the catalyst droplet, the growth of 4H-Si_1–*x*_Ge_*x*_ NWs was initiated with a Ge base. The resulting 4H-Si_1–*x*_Ge_*x*_/Ge NW heterostructure is schematically shown in Figure 2b. Figure S4a presents a HAADF-STEM analysis performed on a NW observed along the ⟨11̅20⟩ zone axis, which clearly reveals the intended SiGe/Ge heterostructure. Figure S4b,c show the Energy Dispersive X-ray (EDX) line profiles for 4H-Si_0.2_Ge_0.8_/Ge and 4H-Si_0.45_Ge_0.55_/Ge NWs, acquired along the NW axis. In the 4H-Si_0.2_Ge_0.8_ segment, the average measured composition is approximately 20% Si and 80% Ge, in agreement with the targeted composition during growth. The relatively abrupt transition between the pure 4H-Ge base and the 4H-Si_1–*x*_Ge_*x*_ alloy sections confirms precise control of the composition upon switching of the precursor gas flows, demonstrating the successful growth of 4H-Si_1–*x*_Ge_*x*_/Ge NWs. The EDX elemental map in Figure S5 shows that the shell regions are slightly Si enriched compared to the axial core.

To confirm the 4H crystal structure in 4H-Si_1–*x*_Ge_*x*_, Figure S6 reveals that the 4H phase, characterized by the ABCB stacking sequence along the *c*-axis ⟨0001⟩, is preserved. However, stacking faults appear between certain 4H segments, which can be attributed to the crystalline quality of the GaAs buffer layer. As previously observed for Ge NWs,^26^ tapering is detected due to lateral growth (see Figure S6) of the cubic phase on the (0001) sidewalls.

We present the macro-PL (see Methods) spectra of our 4H-Ge NWs in Figure 3a with a PL-emission line centered at 0.63 eV. Using the Lasher–Stern–Würfel analysis^17^ (see Figure S7), the bandgap for 4H-Ge is determined at 0.567 ± 0.001 eV, which is 98 meV higher (1 meV lower) than the theoretical predictions based on the MBJLDA (HSE06) functionals,^3^ respectively. The PL-spectra at the highest excitation densities show a small emission peak of the 2H-Ge polytype^1^ around 0.34 eV. The observed PL-spectra are reproducible for a second sample shown in Figure S8.

**Figure 3:**
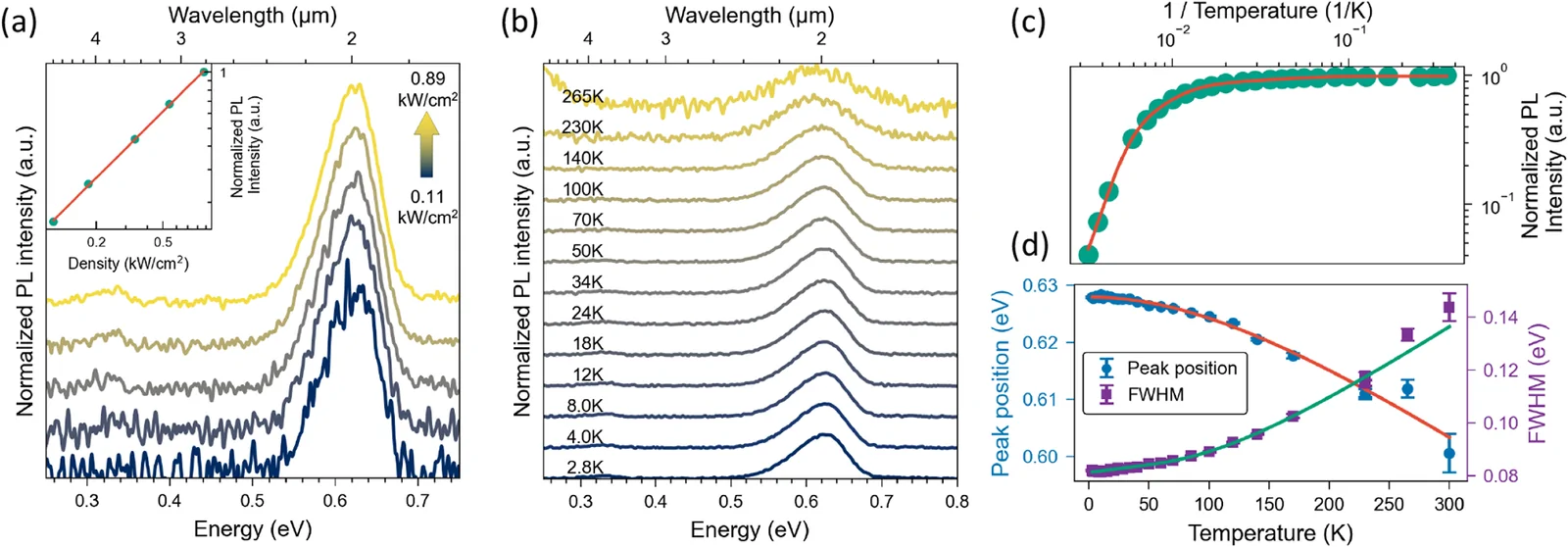
Optical characterization of as-grown 4H-Ge nanowires. (a) Photoluminescence spectra at 4 K as a function of excitation density (0.11–0.89 kW cm^–2^). The excitation densities for the individual spectra are provided in the inset and each spectrum is normalized to its own maximum. The inset shows the integrated photoluminescence intensity versus excitation density on a log–log scale (light-in–light-out curve). The red solid line is a linear fit with slope 0.90 ± 0.01. (b) Normalized photoluminescence spectra measured as a function of temperature at the highest excitation density (0.89 kW cm^–2^). The 265 K spectrum shows an increasing intensity at low photon energies due to blackbody radiation. (c) Integrated photoluminescence intensity versus temperature in an Arrhenius representation; the red solid line is a fit using a three-term Arrhenius model. (d) Photoluminescence peak energy (blue circles) and full width at half-maximum (purple squares) versus temperature, extracted by fitting the spectra in (b) with an asymmetric Gaussian. The red and green solid lines are fits using a model for the shift of the bandgap with temperature and a model for the photoluminescence line width, respectively.

The inset of Figure 3a shows the light-in–light-out plot^1,30^ on a log–log scale recorded at 4 K, showing a slope of 0.90 ± 0.01. This plot is expected to provide a slope of unity for pure radiative recombination, a slope of two for Shockley–Read–Hall recombination, and a slope of 2/3 for Auger recombination. The slope of 0.90 ± 0.01 indicates almost pure radiative recombination and no clear sign of Shockley–Read–Hall recombination.

As a next step, we transferred individual 4H-Ge NWs to a Si/SiO_2_ substrate and performed micro-PL measurements on single NWs (see Figure S9 and Methods). The micro-PL spectra are similar to those obtained in macro-PL on a 4H-ZnS substrate, excluding the possibility that the observed PL in macro-PL is not originating from the 4H-Ge NWs themselves.

The temperature dependence of the PL spectra is shown in Figure 3b. PL is observed up to a temperature of 265 K, which is quite surprising for a parity-forbidden transition and points toward low nonradiative recombination losses. We observe a clear redshift (Figure 3b) of the PL-spectrum, which is attributed to the well-known Varshni shift of the bandgap, providing evidence that the observed PL is related to the 4H-Ge bandgap. Moreover, the high temperature (*T* ≥ 140 K) PL-spectra in Figure 3b clearly show a broadening of the Fermi–Dirac high energy tails, which is a proof for the observation of band-to-band recombination at elevated temperatures.

The temperature-dependent quenching of the emission in Figure 3c is accurately described by a three-term Arrhenius function,^1^ with nonradiative recombination activation energies of *E*_*A*_ = (3.6 ± 0.4) meV, *E*_*B*_ = (28 ± 2) meV, and *E*_*C*_ = (115 ± 8) meV, with corresponding quenching rates of *R*_*A*_ = 0.27 ± 0.04, *R*_*B*_ = 8 ± 2, and *R*_*C*_ = (1.5 ± 0.5) × 10^3^. The Arrhenius plot shows that the emission intensity is highest at low temperature and remains relatively constant up to 100 K, providing experimental proof for a direct bandgap transition, since an indirect bandgap transition causes a decreasing emission toward low temperature,^31^ opposite to what we observe here.

We subsequently obtain a meaningful Debye temperature of Θ = 400 K for 4H-Ge, corresponding to an optical phonon energy of *k*_*B*_Θ = (33 ± 9) meV, by fitting (see Figure S10) the observed monotonic decrease of the 4H-Ge bandgap with increasing temperature in Figure 3d. This value is close to the values obtained for III–V semiconductors,^32^ and becomes even more meaningful by comparing it with the ℏω_LO_ = (33 ± 3) meV phonon energy obtained by fitting (see Figure S10) the temperature dependence of the Full-Width at Half-Maximum of the PL-emission line in Figure 3d. Importantly, these phonon energies are mutually consistent. The predicted phonon energies for 4H-Ge (see Table S3) are similar to the measured phonon energies by Raman spectroscopy for the 2H phase^33^ of 35.7 and 37 meV. Our phonon energy for 4H-Ge is finally consistent with the range of 30 to 38 meV predicted by theory.^23,25^

To verify the tunability of the emission energy with Ge-composition, we experimentally study the optical properties of 4H-Si_1–*x*_Ge_*x*_. Macro-PL spectra of NWs with a 4H-Ge bottom section and a 4H-Si_0.2_Ge_0.8_ top section are presented in Figure 4a using the fitting procedure explained in Figure S11. At low temperature, carriers initially photoexcited into the 4H-Si_0.2_Ge_0.8_ top section will remain localized at alloy fluctuations, without diffusing toward the low bandgap 4H-Ge bottom section. We thus observe two distinct emission peaks: one peak centered at 0.63 eV originating from the 4H-Ge segment (which matches the behavior shown in Figure 3a) and a higher-energy peak from the higher bandgap 4H-Si_0.2_Ge_0.8_ segment with a maximum at 0.74 eV. A Lasher–Stern–Würfel analysis^17^ as shown in Figure S11b, yields bandgaps of 0.577 ± 0.001 eV and 0.691 ± 0.001 eV for 4H-Ge and 4H-Si_0.2_Ge_0.8_, respectively. An overview of the measured physical properties of 4H-Ge is presented in Table 1. In Figure S12, the PL-spectra of these NWs are plotted as a function of excitation density, together with the light-in–light-out graph^1,30^ which exhibits a slope of 0.81 ± 0.01 for the 4H-Si_0.2_Ge_0.8_ part of the emission, similar to 4H-Ge.

**Figure 4:**
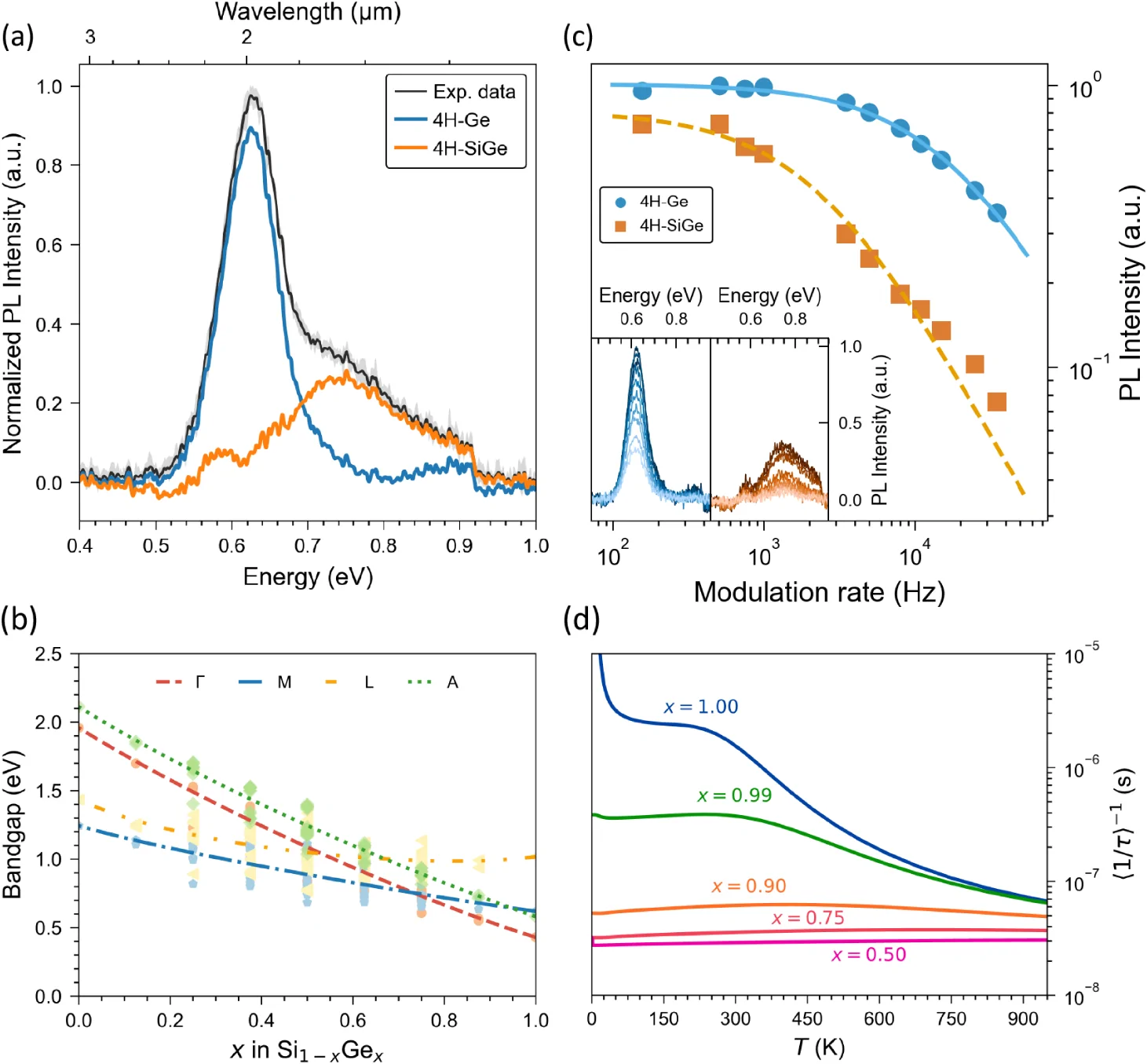
Photoluminescence spectrum and recombination lifetime of as-grown 4H-SiGe nanowires, combined with the theoretical results. (a) Photoluminescence spectrum (black) of 4H-Si_0.2_Ge_0.8_ nanowires taken at 4K. The photoluminescence spectrum can be decomposed into emission spectra due to 4H-Ge (blue) and 4H-Si_0.2_Ge_0.8_ (orange). (b) Band ordering of the four lowest conduction bands in 4H-Si_1–*x*_Ge*_x_* alloys, showing a direct bandgap for Ge compositions above 73%. The individual data points are calculated by density functional theory for all symmetry-inequivalent atomic configurations of the 4H unit cell, while the lines are composition averages using the strict-regular solution model (see Methods). (c) Photoluminescence measurements as a function of the modulation frequency, showing an effective recombination lifetime of 400 ± 50 μs (orange) for 4H-Si_0.2_Ge_0.8_ and of 55 ± 3 μs (blue) for 4H-Ge. The inset shows the 4H-Ge part (blue) and the 4H-Si_0.2_Ge_0.8_ part (orange) of the photoluminescence spectra acquired at modulation frequencies of the excitation laser indicated in the main panel. The integrated spectra are fitted to a low-pass filter model (eq 1). (d) Theoretical results for the radiative lifetime of the 4H-Si_1–*x*_Ge*_x_* alloys, as a function of temperature. The radiative lifetime is calculated as the inverse of the thermal average of the recombination rates at temperature *T* (see Methods). The Ge-composition is indicated at each graph.

**Table 1:** Physical Properties of 4H-Ge Obtained in This Work, Compared to Theory (This Work and Literature) and Experimental Values for 2H-Ge

Parameter	Symbol	Experimental 4H-Ge	Theory 4H-Ge	Experimental 2H-Ge
Bandgap	*E*_*g*_	0.567 ± 0.001 eV	0.469–0.568 eV (MBJLDA-HSE06)	0.353 eV^1^
Debye temperature	Θ	400 ± 100 K	—	66 K^1^
Phonon energy	*k*_*B*_Θ	33 ± 9 meV	—	35.7–37 meV^33^
Longitudinal optical phonon energy	ℏω_LO_	33 ± 3 meV	30–38 meV^23,25^	—
Activation energies	*E*_*A*_	3.6 ± 0.4 meV	—	16 ± 1 meV^1^
	*E*_*B*_	28 ± 2 meV	—	91 ± 2 meV^1^
	*E*_*C*_	115 ± 8 meV	—	—
Light-in–light-out slope	*k*	0.90 ± 0.01	—	1.02 ± 0.02 ^1^
PL lifetime	τ	55 ± 3 *μ*s	—	2.1 ± 0.3 ns^17^

To place the experimental observations in a theoretical context, we performed *ab initio* DFT calculations (see Methods) of the electronic band structure for 4H-Si_1–*x*_Ge_*x*_ as shown in Figure 4b. Importantly, the 4H polytypes feature a direct bandgap for 4H-Si_1–*x*_Ge_*x*_ with *x* ≥ 0.73. Taking into account that the theoretical bandgap of 4H-Ge is overestimated (underestimated) by 1 meV (98 meV) by the HSE06 (MBJLDA) functionals, and assuming the same corrections for 4H-Si_0.2_Ge_0.8_, the expected bandgap for 4H-Si_0.2_Ge_0.8_ is between 0.67 eV (HSE06) and 0.77 eV (MBJLDA), while experiment yields a bandgap of 0.691 ± 0.001 eV for 4H-Si_0.2_Ge_0.8_. We obtain excellent agreement between theory and experiment for the HSE06 functional.

To investigate the recombination lifetime in 4H-Ge, we first performed time-resolved micro-PL measurements (see Methods). The time-resolved spectral PL map in Figure S13a essentially shows no decay. A recombination lifetime ≫ 20 ns is in full agreement with theory^3^ and much longer than the reported lifetimes ∼1 ns in 2H-Ge and 2H-SiGe.^1,17^ The dynamics immediately after excitation (*t* ≤ 0.5 ns) is further discussed in Figure S14.

To quantitatively obtain the recombination lifetime of 4H-Ge and 4H-Si_0.2_Ge_0.8_, we measured the dependence of the demodulated quasi-continuous wave (quasi-CW) PL signal on the modulation frequency of the excitation laser (see Methods). The left (right) inset of Figure 4c shows the PL-spectra of the bottom Ge (top SiGe) section in blue (orange) of the 4H-Si_0.2_Ge_0.8_/Ge NWs, acquired as a function of modulation frequency. The integrated PL intensity versus the modulation rate in Figure 4c shows a first-order low-pass response (see Methods) that yields a recombination time constant τ = 55 ± 3 μs for 4H-Ge and τ = 400 ± 50 μs for 4H-Si_0.2_Ge_0.8_. For the pure 4H-Ge sample shown in Figure S13b,c, we obtain a similar recombination time constant of τ = 120 ± 10 μs. Given the very long effective recombination lifetimes observed (1/τ_*effective*_ = 1/τ*_radiative_* + 1/τ_*nonradiative*_), it is very surprising that we are able to observe detectable photoluminescence on the best parts of the sample using infrared photodetectors with limited sensitivity (see Methods). Since we measure the effective recombination time, this means that the radiative and nonradiative lifetimes in the 4H-Ge section of the 4H-Si_0.2_Ge_0.8_/Ge NWs should each individually be greater than (55 ± 3) μs. This implies that both the Shockley–Read–Hall (SRH) recombination lifetime and the Auger recombination lifetime in 4H-Ge are larger than (55 ± 3) μs under our experimental conditions. Long effective lifetimes are possible because surface recombination in our 4H-Ge and 4H-SiGe NWs is reduced due to passivation by the cubic shell, providing type I band alignment between core and shell.^3^

The radiative lifetimes for 4H-Si_1–*x*_Ge_*x*_/Ge in the Ge-rich composition range down to *x* = 0.50 (Figure 4d) were calculated by *ab initio* DFT from the thermally averaged optical transition matrix elements. Due to the parity-forbidden character of the Γ → Γ transition, τ diverges strongly as T → 0, since at low carrier temperature only the lowest-lying, optically forbidden states are thermally populated. This strong temperature dependence is a direct fingerprint of the parity-forbidden transition and clearly distinguishes 4H-Ge from its 2H counterpart. A small addition of Si already breaks the inversion symmetry of the pure 4H-Ge lattice, lifting the parity selection rule and reducing the lifetime by several orders of magnitude, as seen for x = 0.99 in Figure 4d.

In contrast to 2H-Ge,^17^ theory and experiment in 4H-Ge are relatively close together, since theory predicts a radiative lifetime of >10 μs for 4H-Ge at low temperature, while experiment measures effective lifetimes of 55 μs for the Ge section of the 4H-Si_0.2_Ge_0.8_/Ge NW and 120 μs for pure 4H-Ge. When comparing theory with experiment, it is important to realize that the relevant temperature quoted in theoretical papers for the radiative lifetime is, in fact, the carrier temperature, which is often relatively high in optical experiments.^34^ In addition, since the coupling of the dipoles to the *HE*_11_ mode becomes very weak for our thin 4H-Ge nanowires, spontaneous emission might be partially inhibited, resulting in a reported radiative lifetime which is longer than in a bulk crystal.^35,36^ The longer effective lifetime of 400 μs in 4H-SiGe compared to the 55 μs lifetime in 4H-Ge is currently not understood. Unfortunately, the small radiative lifetimes for 4H-Si_1–*x*_Ge_*x*_ as shown in Figure 4d are not experimentally confirmed. On the other hand, the theoretical trend of a very small but finite transition matrix element for 2H-Ge^8^ and a parity-forbidden transition for 4H-Ge^3^ is at least qualitatively confirmed by the experimental radiative lifetimes of 2.1 ns and >55 μs for 2H-Ge and 4H-Ge, respectively.

In conclusion, we report pure 4H-Ge and composition controlled 4H-Si_1–*x*_Ge_*x*_/Ge NWs with high crystal phase purity. The growth of vertical NWs was established by robust crystal structure transfer using the m-plane of a 4H-ZnS substrate and an intermediate 4H-GaAs template. The high crystal phase purity was confirmed by high resolution STEM. *Ab initio* DFT obtains radiative lifetimes of >10 μs and 32 ns at low temperature for 4H-Ge and 4H-Si_0.25_Ge_0.75_ respectively, for the parity-forbidden fundamental transition. Despite the >10 μs recombination lifetime, our 4H-Ge NWs emit direct bandgap PL, which is clearly bandgap related and survives up to 265 K. Moreover, the emission energy is in good agreement with *ab initio* DFT calculations. We subsequently demonstrate composition tuning of the emission energy by studying 4H-Si_0.2_Ge_0.8_/Ge heterostructure NWs. We extract effective recombination lifetimes of 55 and 400 μs for 4H-Ge and 4H-Si_0.2_Ge_0.8_. Direct bandgap light emission in 4H-Si_1–*x*_Ge_*x*_ NWs is possible because the nonradiative recombination rate is comparable to the radiative one. Compared to III–V compound semiconductors, 4H-Ge features a remarkably long SRH lifetime, approaching the extremely long SRH lifetimes in cubic Si^37^ and Ge.^38^ This implies a low concentration of midgap impurity levels and point defects, which are primarily responsible for the trapping of photoexcited charge carriers.

## Methods

### Synthesis of 4H-Ge and 4H-Si_1–*x*_Ge*_x_* NWs

For the growth of 4H-Ge NWs, a GaAs buffer layer deposited on an m-plane ZnS substrate was used as a template. All growth processes were carried out in an ultrahigh vacuum vapor phase epitaxy (UHV-VPE) reactor. Prior to growth, the ZnS substrates were prepared by chemical-mechanical polishing using a 0.01% bromine–methanol solution. This treatment, specifically developed for II–VI substrates,^39^ eliminates the need for high-temperature desorption and thereby prevents any increase in surface roughness. Subsequently, the substrates were annealed at 500°C for 1 h under UHV to remove residual bromine contamination. The temperature was then raised to 550°C under a TBAs flux of 30 sccm (P = 5 × 10^–3^ Torr) and stabilized for 5 min. GaAs growth was initiated at 550°C by introducing trimethylgallium (TMGa) at a flow rate of 3 sccm, corresponding to a V/III ratio of 10, and was maintained for 2 min at the same pressure. Ge NWs were synthesized via the VLS growth mechanism using Au as a catalyst. A thin Au film was deposited on the GaAs buffer layer by electron-beam evaporation under UHV conditions and subsequently dewetted at 400°C in the growth chamber to form catalytic droplets with a diameter of approximately 30 nm. Ge NW growth was then carried out at 350°C using digermane (Ge_2_H_6_) as the precursor, with a flow rate of 2 sccm and a pressure of 5 × 10^–2^ Torr. The growth of 4H-SiGe/Ge NWs was initiated by first forming a Ge stem to stabilize the Au catalyst droplet. This step is essential because the introduction of Si during growth was found to induce significant droplet instability on the substrate under our conditions. The Ge base was therefore grown under the same conditions as those used for 4H-Ge NWs to ensure a stable catalyst configuration prior to alloy growth. The temperature was then progressively increased to 400°C under simultaneous silane (SiH_4_) and germane (GeH_4_) flows at a constant pressure of 5 × 10^–2^ Torr, with a ramp duration of 12 min. For the growth of the SiGe segment, constant gas flows of 16 sccm GeH_4_ and 4 sccm SiH_4_ were used, aiming at a nominal composition of 20% Si and 80% Ge. In contrast to the Ge NW growth, digermane was replaced by germane in order to achieve better control of the Si/Ge ratio. This choice also ensures more comparable decomposition kinetics between GeH_4_ and SiH_4_, facilitating more uniform alloy incorporation. The 4H-Si_0.2_Ge_0.8_/Ge heterostructure was subsequently grown for 15 min.

### Photoluminescence Measurements

The as-grown 4H-Ge NWs are characterized using quasi-continuous wave (quasi-CW) macro-PL as well as by time-resolved micro-PL techniques.

The quasi-CW macro-PL measurements are performed using a Fourier transform infrared spectroscopy (FTIR) setup. A 976 nm laser modulated at 500 Hz excites the sample with a 100 μm spot size, enabling ensemble measurements of numerous NWs. The collected PL is spectrally resolved using a Thermo Scientific Nicolet iS50R FTIR interferometer and detected with a built-in mercury cadmium telluride detector combined with a lock-in amplifier to suppress spurious signal contributions.

NW lifetimes are studied using a time-resolved FTIR setup employing a pulsed laser (1030 nm, 200 fs pulse duration, 40 MHz repetition rate). The excitation laser is focused onto the as-grown NWs using a 36× magnification, NA = 0.52 Cassegrain objective, yielding a 4 μm spot size. The laser fluence is maintained at 2 mJ cm^–2^ throughout this experiment. This configuration also enables single NW measurements, as discussed in Figure S9. The emitted PL is collected and spectrally resolved by a Nireos Gemini interferometer module, while lifetime measurements are performed via Time-Correlated Single Photon Counting using a PicoQuant PicoHarp 2000 system coupled with a Superconducting NW Single Photon Counter. This configuration enables lifetime measurements up to 20 ns.

For longer lifetimes exceeding the 20 ns measurement window of the time-resolved FTIR setup, we employ an alternative approach based on the quasi-CW PL measurements. Specifically, we monitor the demodulated PL signal from the lock-in amplifier as a function of laser modulation frequency. Because we use synchronous detection with a lock-in amplifier, we expect that if carriers have not completely recombined before the next modulation pulse arrives, the measured AC signal will be reduced as the baseline increases. This behavior can be modeled as a low-pass filter, where the frequency response is given by

1 $$\frac{I\left(f\right)}{I_{0}}=\frac{f_{0}}{f_{0}+f}$$

where *I*_0_ represents the intensity at zero modulation frequency (DC limit) and *f*_0_ is the cutoff frequency. The effective carrier recombination time constant is then extracted as $\tau =\frac{1}{f_{0}}$ .

### *Ab Initio* Calculations

Electronic band structures were computed using the Vienna *Ab initio* Simulation Package (VASP)^40,41^ within the projector-augmented wave formalism, employing the modified Becke–Johnson exchange-correlation potential^42^ with spin–orbit coupling to accurately describe band gaps and optical matrix elements.^3^ The composition dependent properties of 4H-Si_1–*x*_Ge_*x*_ alloys were obtained by thermodynamic averages over all symmetry-inequivalent atomic configurations of the 4H unit cell, following the strict regular-solution (SRS) model.^11,43,44^ Radiative lifetimes were computed by thermally averaging the radiative recombination rate over vertical optical transitions, using dense and complementary Monkhorst–Pack grids in reciprocal space to ensure convergence. Full computational details are provided in the Supporting Information.

## Supplementary Material



## Notes

Concept of 4H-Ge NW growth; NW structure and orientation; bright field STEM image of 4H-Ge; composition control and EDX analysis of 4H-Si_1–x_Ge_x_ NWs; bright field STEM image of a 4H-SiGe NW; Lasher–Stern–Würfel fit; second 4H-Ge sample; PL spectra from individual NWs; spectral line shape analysis; phonon temperature of 4H-Ge; optical phonon energy of 4H-Ge; PL of 4H-SiGe; time resolved PL of 4H-Ge; initial PL decay; *ab initio* calculations (PDF)
