# Supplementary material for: Crystal Phase Engineering and Light Emission from 4H-SiGe Nanowires
Source: ACS Nanosci Au. 2026 Aug 19;6(5):895–902. doi: 10.1021/acsnanoscienceau.6c00062 (PMC13693219; doi:10.1021/acsnanoscienceau.6c00062)
Supplement: Supplementary file 1 [file ng6c00062_si_001.pdf]

Crystal phase engineering and light emission from 4H-SiGe nanowires

Riccardo Farina,[†,‖] Hafssa Ameziane,[‡,‖] Victor van Lange,[†,‖] Pedro Borlido,[¶,‖] Gilles Patriarche,[‡] Charles Renard,[‡] Erik P.A.M. Bakkers,[†] Silvana Botti,[§] Laetitia Vincent,[*,‡] and Jos E.M. Haverkort[*,†]

†*Eindhoven University of Technology, De Groene Loper 19, Eindhoven 5612 AZ, The Netherlands*

‡*Université Paris-Saclay, CNRS, Centre de Nanosciences et de Nanotechnologies, F-91120 Palaiseau, France*

¶*CFisUC, Department of Physics, University of Coimbra, Rua Larga, 3004-516 Coimbra, Portugal*

§*Research Center Future Energy Materials and Systems, Interdisciplinary Centre for Advanced Materials Simulation, Faculty of Physics and Astronomy, Ruhr University Bochum, Universitätsstrasse 150, 44801 Bochum, Germany*

‖*Equal contribution*

E-mail: Laetitia.vincent@cnrs.fr; j.e.m.haverkort@tue.nl

Concept of 4H-Ge NW growth

The direct VLS growth of 4H-Ge and 4H-Si NWs on sulfurized ZnS and CdS substrates, without using a 4H-GaAs pseudo substrate, resulted in inclined NWs or, more frequently, crawling structures along the substrate surface. This behavior is attributed to the instability of the Au catalyst droplet on the substrate surface. Upon exposure to the precursors, the

droplet tends to migrate, leading to uncontrolled catalyst-assisted growth. As a result, all NWs exhibited the conventional cubic crystal structure. To overcome these limitations, surface patterning strategies should be implemented to improve catalyst droplet stabilization and localization. Furthermore, increasing the growth pressure would enhance precursor partial pressures and, consequently, the supersaturation within the Au droplet, thereby promoting axial growth and reducing the tendency for catalyst migration. Given that GaAs-2H NWs have already demonstrated the feasibility of growing Ge nanobranches on their sidewalls without noticeable difficulties,[1] this work therefore proposes the use of GaAs buffer layers as templates for Ge NW growth. Such an approach is expected to provide improved anchoring of the Au catalyst droplet and more favorable conditions for controlled axial growth. Although the synthesis of 4H-GaAs epilayers has not been reported before, it is beyond the scope of this paper.

4H-Ge NW structure and orientation

The Ge NWs were grown using the VLS mechanism employing Au catalyst droplets on a m-plane $(1\bar{1}00)$ oriented substrate. As shown in Fig. 2e and discussed in the main text, the stabilization of the hexagonal crystal phase requires vertically oriented NWs, grown along the $\langle 1\bar{1}00 \rangle$ direction. Fig. S1 shows top-view SEM images collected from two different samples and two different zones of the m-plane 4H-GaAs/4H-ZnS template. In these images, bright circular spots correspond to vertical NWs, while the linear contrast corresponds to inclined NWs, which are expected to have the cubic crystal phase. A small fraction of NWs exhibit a noticeable inclination with a preferential orientation toward either the $\langle 0001 \rangle$ or the $\langle 11\bar{2}0 \rangle$ direction. The corresponding surface density and vertical/inclined ratio are summarized in Table S1. Although the total NW density varies between the investigated zones, the most relevant parameter for stabilizing the hexagonal phase is the density and fraction of vertical NWs. Table S1 shows that the regions with the highest total NW density do not necessarily

correspond to the highest fraction of vertical NWs. In particular, Sample 1 exhibits a larger fraction of vertical NWs, with fractions of 74% and 58% for Zones 1 and 2, respectively, whiles Sample 2 contains a higher fraction of inclined NWs, with vertical fractions of only 41% and 30% for Zones 1 and 2, respectively. The formation of inclined NWs, as well as the variation in the vertical and inclined NW ratio observed between different zones of the same sample and between different samples, is likely related to the quality of the 4H-GaAs buffer. Surface defects in the 4H-GaAs buffer, such as residual roughness, can hinder proper epitaxial alignment with the substrate. These surface irregularities can prevent the establishment of a coherent epitaxial relationship, leading to the formation of tilted NWs with a non-epitaxial standard cubic crystal structure. These results highlight the key role of buffer layer quality in controlling 4H-Ge NW growth and specifically the orientation of the NWs. We believe that the spatial inhomogeneity of the photoluminescence (PL) emission observed across the sample, where some regions exhibit light emission while others do not, reflects variations in the ratio of vertical to inclined NWs. This is because the inclined cubic Ge NWs are not expected to contribute to the PL signal.

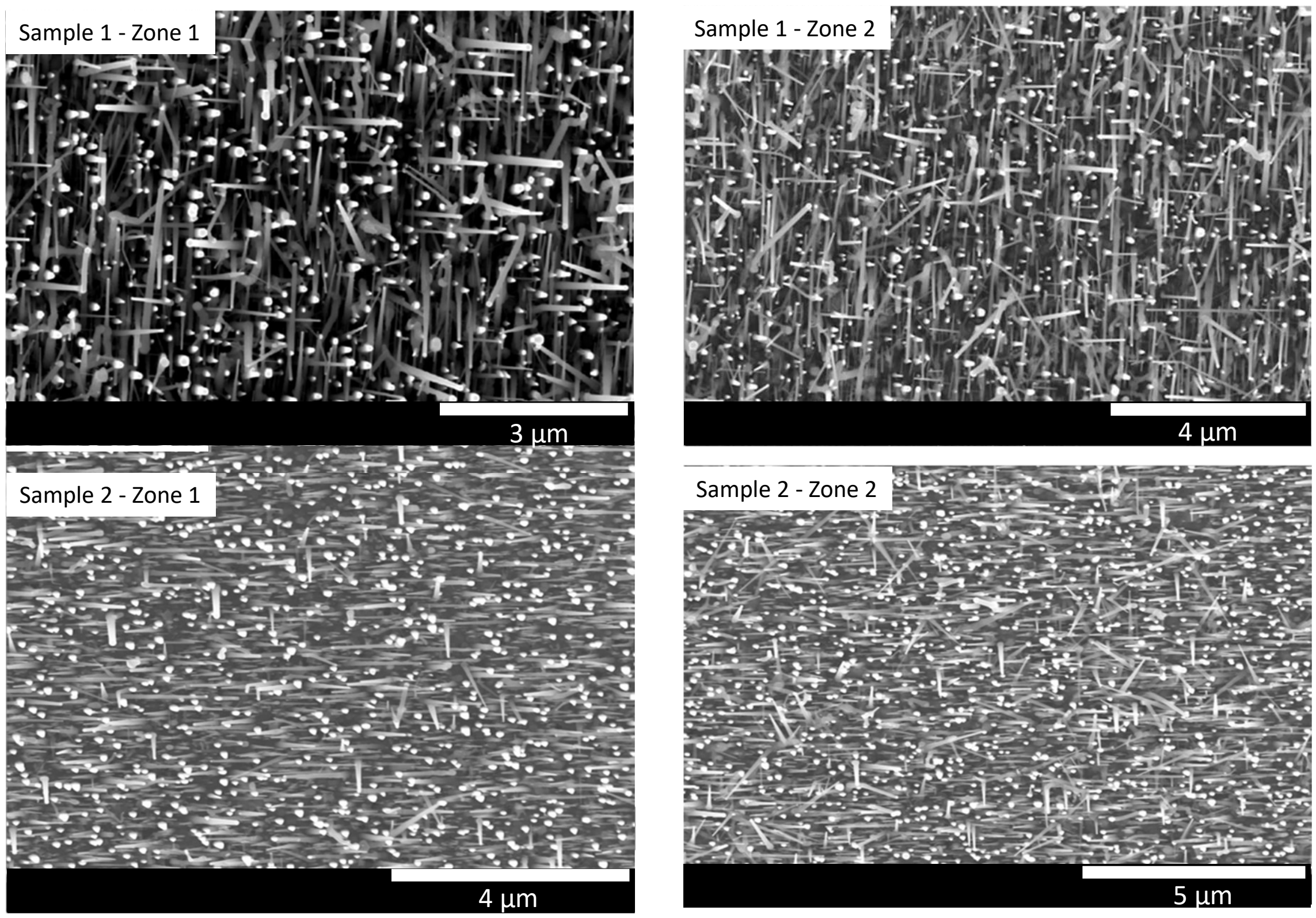

Figure S1: Top-view SEM images of Ge NWs collected from two different samples and two different zones of the m-plane 4H-GaAs/4H-ZnS substrate.

	Surface density (NW/cm^2)	Vertical/inclined ratio
Sample 1 – Zone 1	1×10^9	74/26
Sample 1 – Zone 2	3×10^8	58/42
Sample 2 – Zone 1	8×10^9	41/59
Sample 2 – Zone 2	2.6×10^9	30/70

Table S1: Surface density and ratio of vertical to inclined Ge NWs measured on different samples and zones.

The 4H crystal structure was systematically observed across several tens of NWs dispersed on a carbon grid. Importantly, 3C-Ge and 4H-Ge NWs can be readily distinguished during subsequent individual characterizations based on their cross-sectional geometry, which is hexagonal for the cubic nanowires and square or rectangular for the hexagonal ones. Moreover, within the range of growth conditions optimized in this study (350–380 °C and 5×10^{-2} to 10^{-1} mbar), no significant variation was observed in the hexagonal phase content.

Notably, no I3 defects[2] were observed in the NWs grown in this study, which are known to reduce hexagonality when growing 2H-SiGe NW shells. However, the defect density exhibited substantial variations from one NW to another. These variations are likely related to differences in the crystalline quality of the GaAs pseudo-substrate, whose defect density can locally vary as a result of surface roughness fluctuations.

The tapered morphology observed in Fig. S2a indicates a prolonged contribution of the vapor–solid (VS) growth mechanism at the base of the nanowire relative to its upper region. The enlarged regions highlight the difference between the upper part and the base of the NW.

The HR-STEM image acquired at the NW base in Fig. S2b reveals the presence of lateral cubic 3C-Ge segments along the sidewalls of the main 4H-Ge NW. Their growth direction is along the $\langle 0001 \rangle$ direction of the hexagonal phase, which is equivalent to the $\langle 111 \rangle$ direction in the cubic phase. This indicates that the tapering is associated with a vapor–solid contribution competing with the axial VLS growth. Therefore, the NW morphology results from the coexistence of axial 4H-Ge growth and lateral 3C-Ge growth, particularly near the NW base.

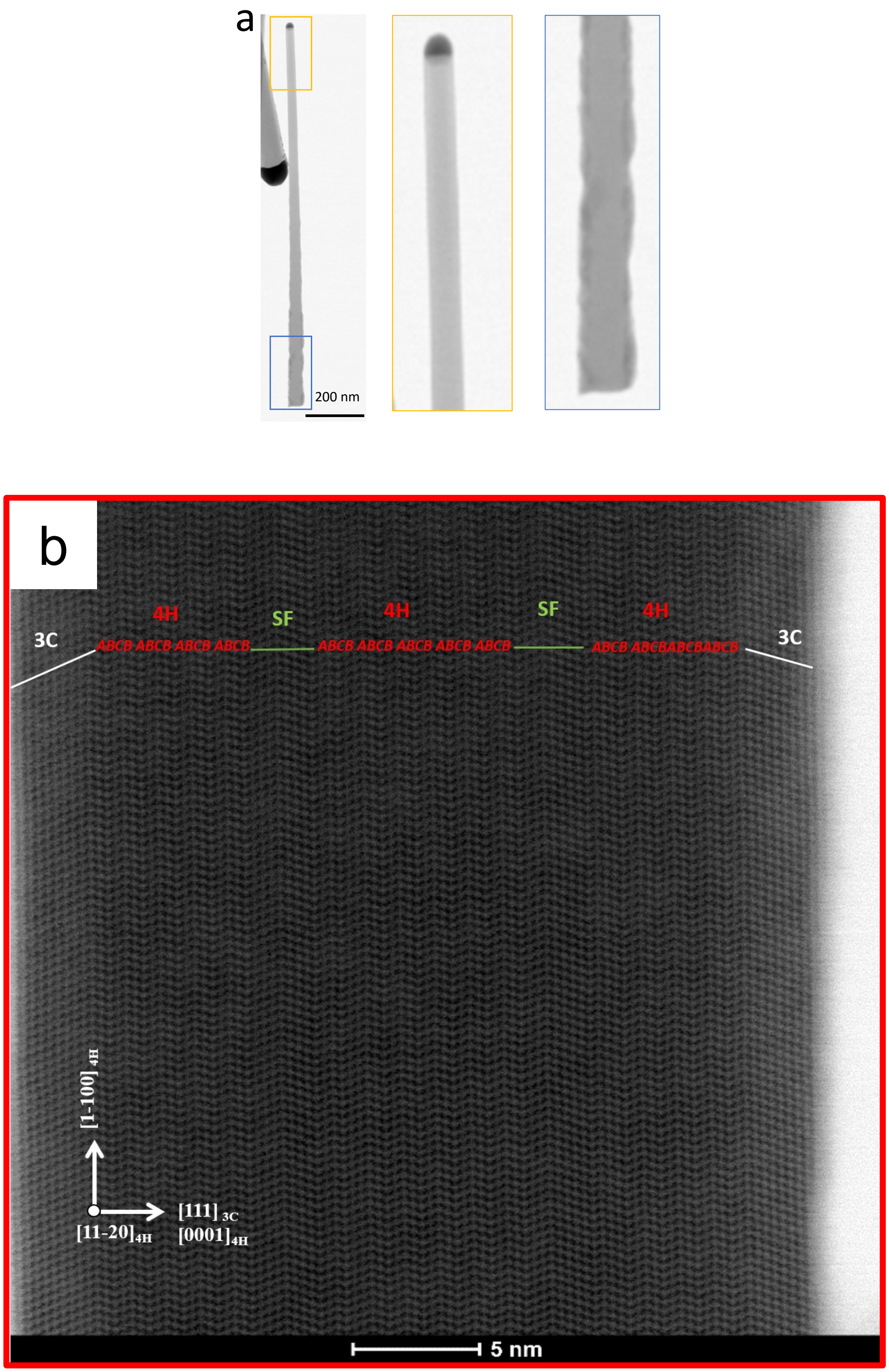

Figure S2: (a) TEM image of an individual 4H-Ge NW, together with enlarged views of the upper part and the base, highlighting the diameter variation along the NW axis. (b) BF-STEM image of a 4H-Ge NW acquired at the NW base, revealing cubic (3C) lateral growth on the sidewalls.

Fig. S3 provides additional structural evidence of the tapering observed in Ge NWs. The SEM image in Fig. S3 shows that the NWs exhibit a tapered morphology, with a larger diameter close to the base and a smaller diameter toward the top. This morphology suggests that, in addition to the axial VLS growth driven by the Au catalyst droplet, a lateral vapor–solid (VS) growth contribution occurs on the NW sidewalls.

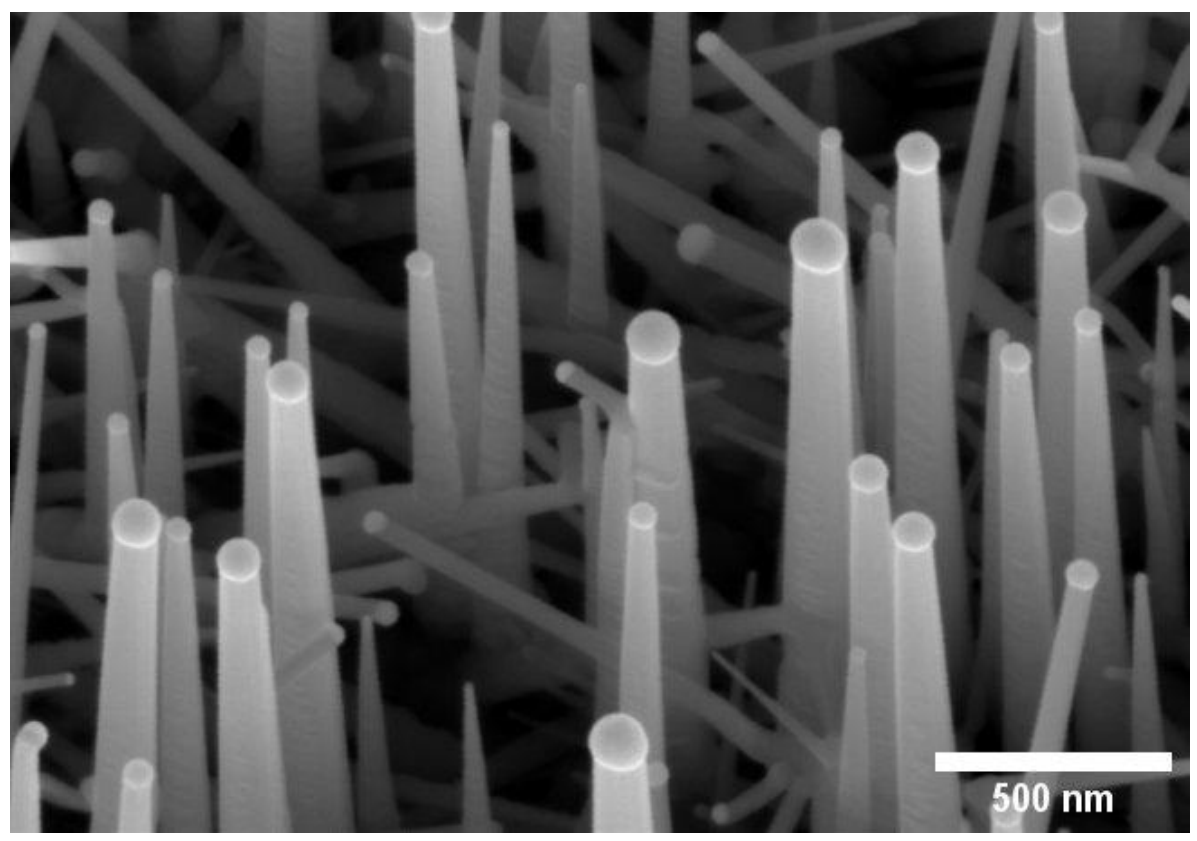

Figure S3: SEM image of Ge NWs showing a tapered morphology, with a larger diameter near the base and a smaller diameter toward the top.

Composition control and EDX analysis of 4H-$Si_{1-x}Ge_x$/Ge NWs

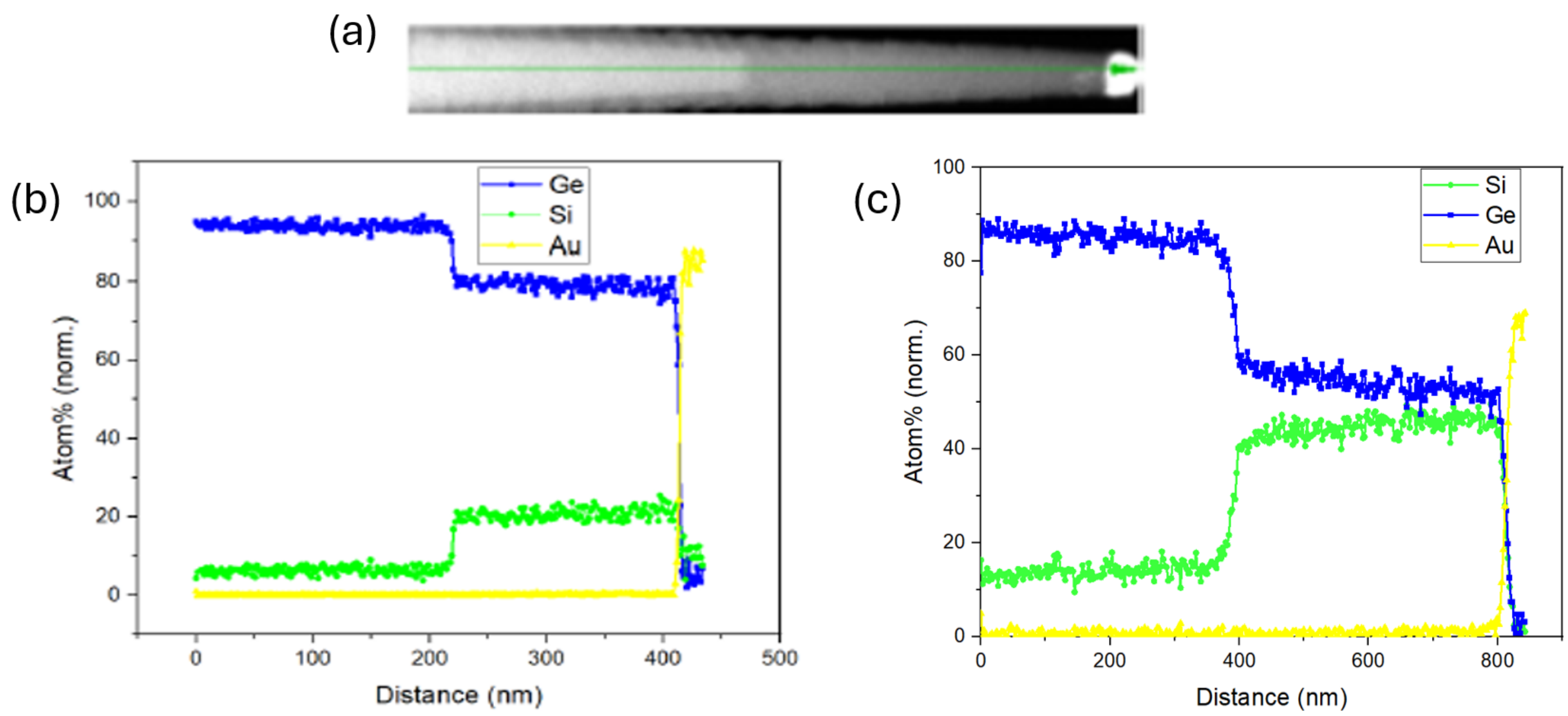

Figure S4: (a) Scanning Transmission Electron Microscopy (STEM) image of the 4H-$Si_{0.45}Ge_{0.55}$/Ge NW. (b) Energy Dispersive X-ray (EDX) analyses showing a Ge-base with a $Si_{0.2}Ge_{0.8}$ top section. (c) EDX analyses showing a Ge-base with a $Si_{0.45}Ge_{0.55}$ top section.

Fig. S4 clearly reveals our ability to grow $Si_{1-x}Ge_x$/Ge heterostructures with different Ge compositions. A germane-to-silane flow rate ratio of 16 to 4 sccm yields a $Si_{0.2}Ge_{0.8}$ composition, while equal flow rates of 10 sccm for both precursors result in a $Si_{0.45}Ge_{0.55}$ alloy. Nevertheless, a gradual stabilization of the composition is observed.

Pronounced tapering is observed, with the NW diameter being larger at the base than at the tip. The presence of a $Si_{1-x}Ge_x$ shell along the NW sidewalls indicates a significant radial growth contribution via the vapor–solid mechanism at relatively high growth temperatures, in addition to axial VLS growth. Notably, this radial overgrowth on the (0001) facets adopts a cubic crystal structure. The EDX elemental map in Fig. S5 shows the spatial distribution of Ge, Si, and Au within the $Si_{1-x}Ge_x$/Ge NW. Au is mainly localized at the catalyst droplet, whereas Ge and Si are distributed along the NW structure, confirming the formation of the $Si_{1-x}Ge_x$ alloy. The local compositions summarized in Table S2 show that the shell regions

are slightly enriched in Si and slightly depleted in Ge compared with the axial core. The Si content increases from about 21.1 at.% in the core to about 24.1–24.6 at.% in the shell, while the Ge content decreases from about 78.9 at.% in the core to about 75.4–75.9 at.% in the shell.

However, considering the 1σ uncertainties reported in Table S2, especially those associated with Ge quantification, the difference between the shell and core compositions is of the same order as the EDX uncertainty. Thus, the observed differences are not sufficient to conclude a significant compositional variation. In addition, the EDX measurement performed in the core region may include a contribution from the shell surrounding the NW. Therefore, the shell and axial core compositions should not be considered strictly identical. Rather, the data suggest comparable $Si_{1-x}Ge_x$ compositions, with a possible slight Si enrichment in the shell.

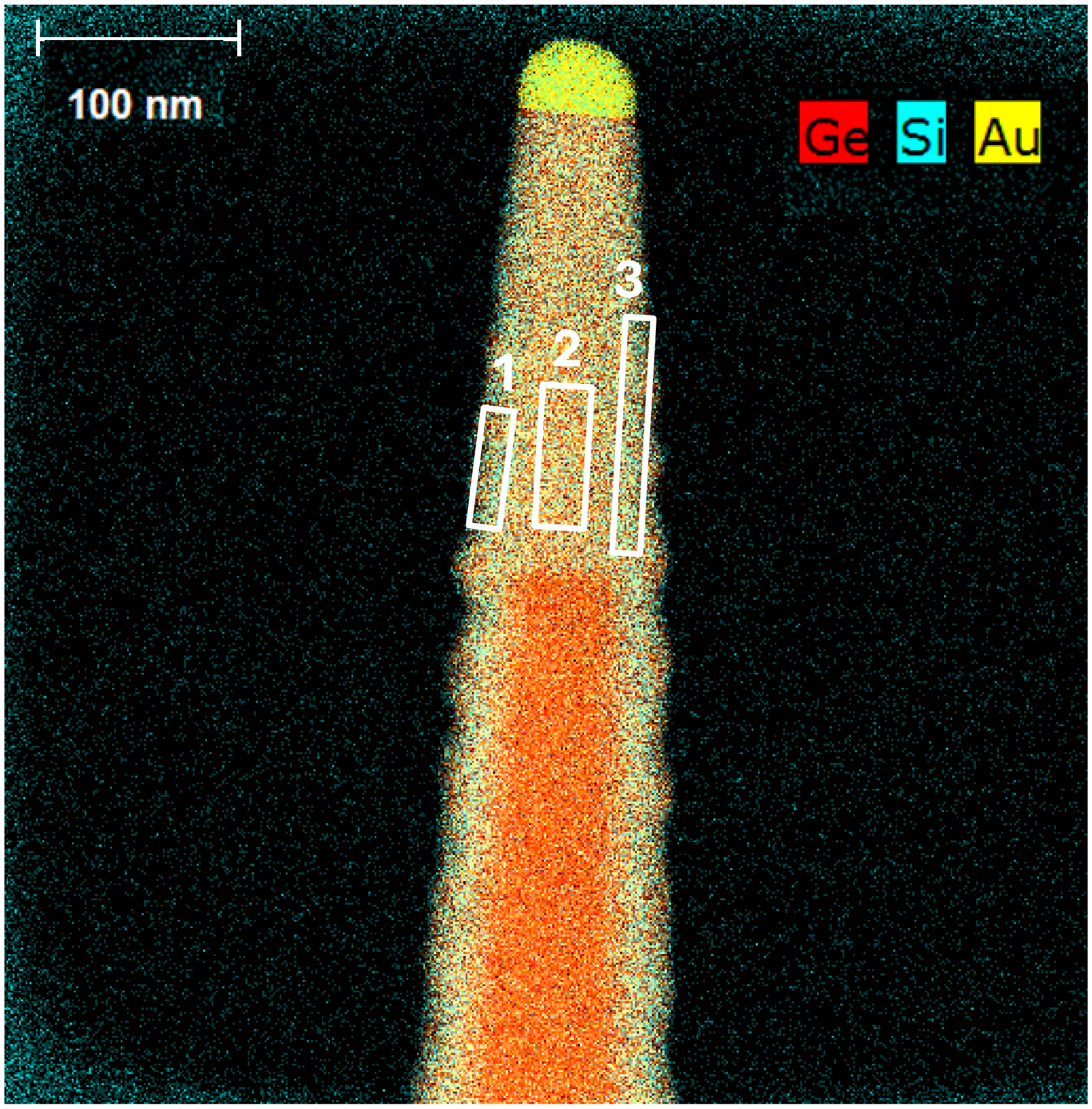

Figure S5: EDX elemental map of a $Si_{1-x}Ge_x$/Ge NW . The numbered regions indicate the areas selected for local composition analysis.

Zone	Region	Si content (at.%)	Ge content (at.%)
Zone 1	Shell	24.07 ± 0.57	75.92 ± 3.41
Zone 2	Core	21.05 ± 0.40	78.94 ± 3.15
Zone 3	Shell	24.56 ± 0.50	75.43 ± 3.23

Table S2: Local EDX composition measured in the axial core and shell regions of the $Si_{1-x}Ge_x$ section. The values are given in atomic percent, and the uncertainties correspond to the 1σ values obtained from the EDX quantification.

Bright field STEM image of a 4H-SiGe NW

BF-STEM analysis in Fig. S6 confirms the predominance of the hexagonal 4H stacking sequence in the $Si_{1-x}Ge_x$ NW section, with only occasional stacking faults, which may originate from structural defects in the 4H-GaAs buffer layer. The image is acquired along the $[11\bar{2}0]_{4H}$ zone axis and shows a NW growing along the $[1\bar{1}00]_{4H}$ direction. In addition, cubic 3C regions are observed on the lateral sidewalls of the NW, indicating the presence of a radial shell contribution. These 3C regions correspond to lateral overgrowth on the (0001) facets, whereas the axial core of the NW preserves the 4H crystal structure. This radial shell is attributed to concurrent vapor–solid (VS) deposition occurring in parallel with axial VLS growth.

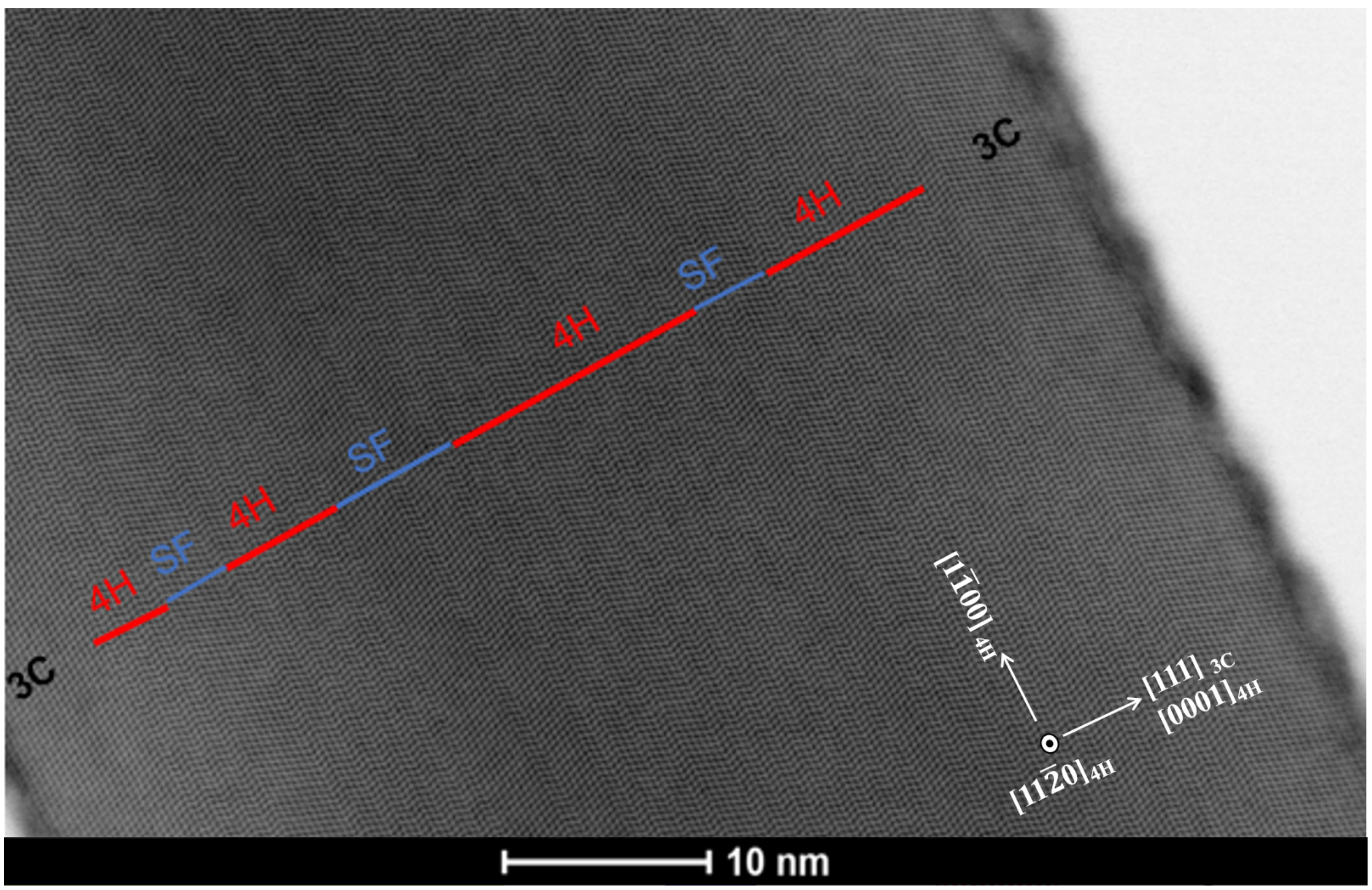

Figure S6: BF-STEM image of a 4H-SiGe NW, acquired along the $\langle 1\bar{1}20\rangle$ zone axis, showing the 4H crystal structure of a NW growing along the $\langle 1\bar{1}00\rangle$ direction, with occasional stacking faults (SF).

Lasher–Stern–Würfel fit

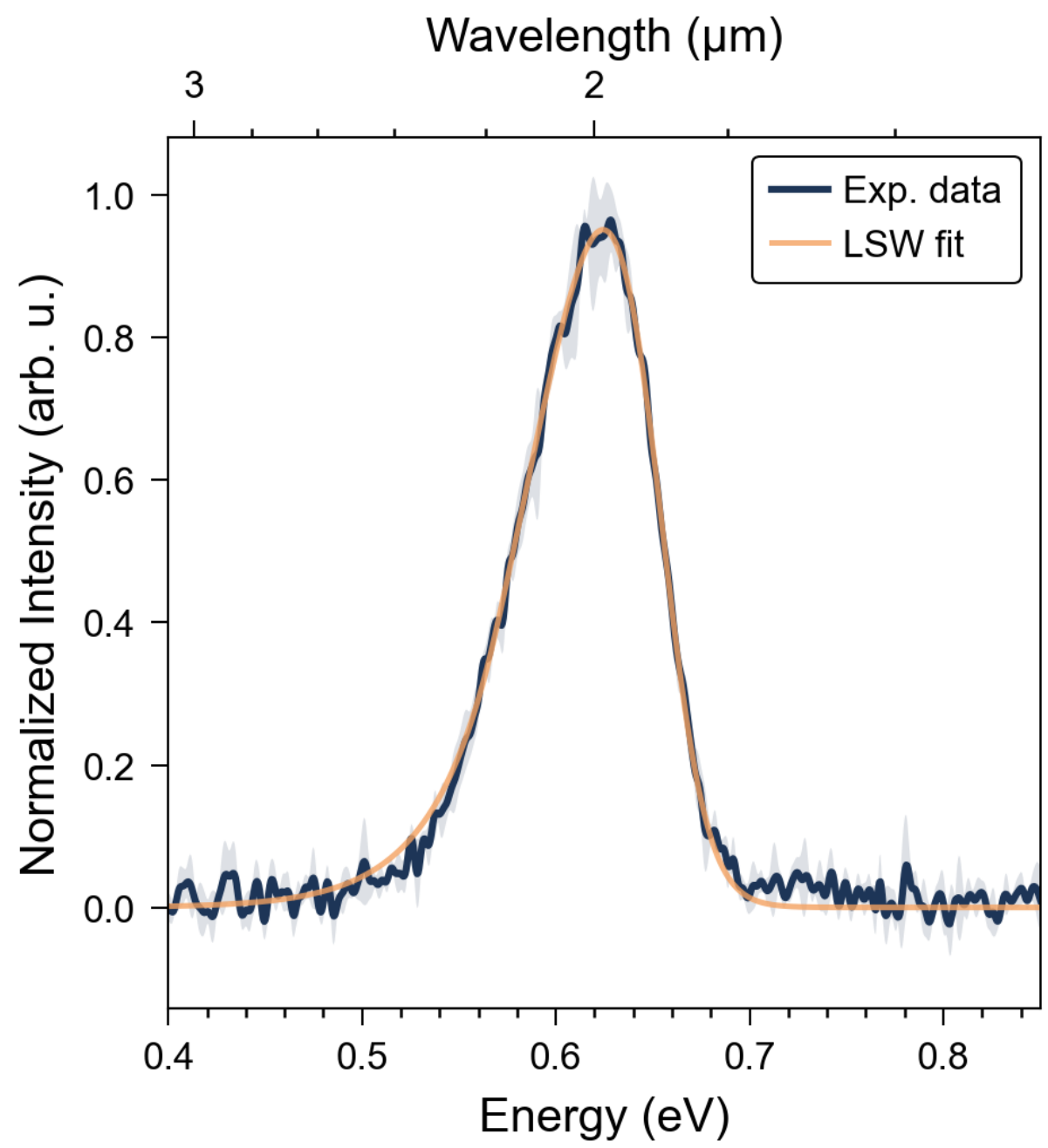

Figure S7: Lasher–Stern–Würfel (LSW) fit of the PL spectrum of 4H-Ge. The LSW model is a generalization of Planck's law for a semiconductor under carrier injection, introducing a non-zero photon chemical potential equal to the quasi-Fermi level splitting and multiplying the resulting emission by the absorption coefficient of the material. The blue solid line is the average spectrum obtained from Fig. 3a in the main text, while the shaded area is the 1-σ interval. The corresponding bandgap extracted from the LSW fit (yellow solid line) for 4H-Ge is 0.567±0.001 eV.

Second 4H-Ge sample

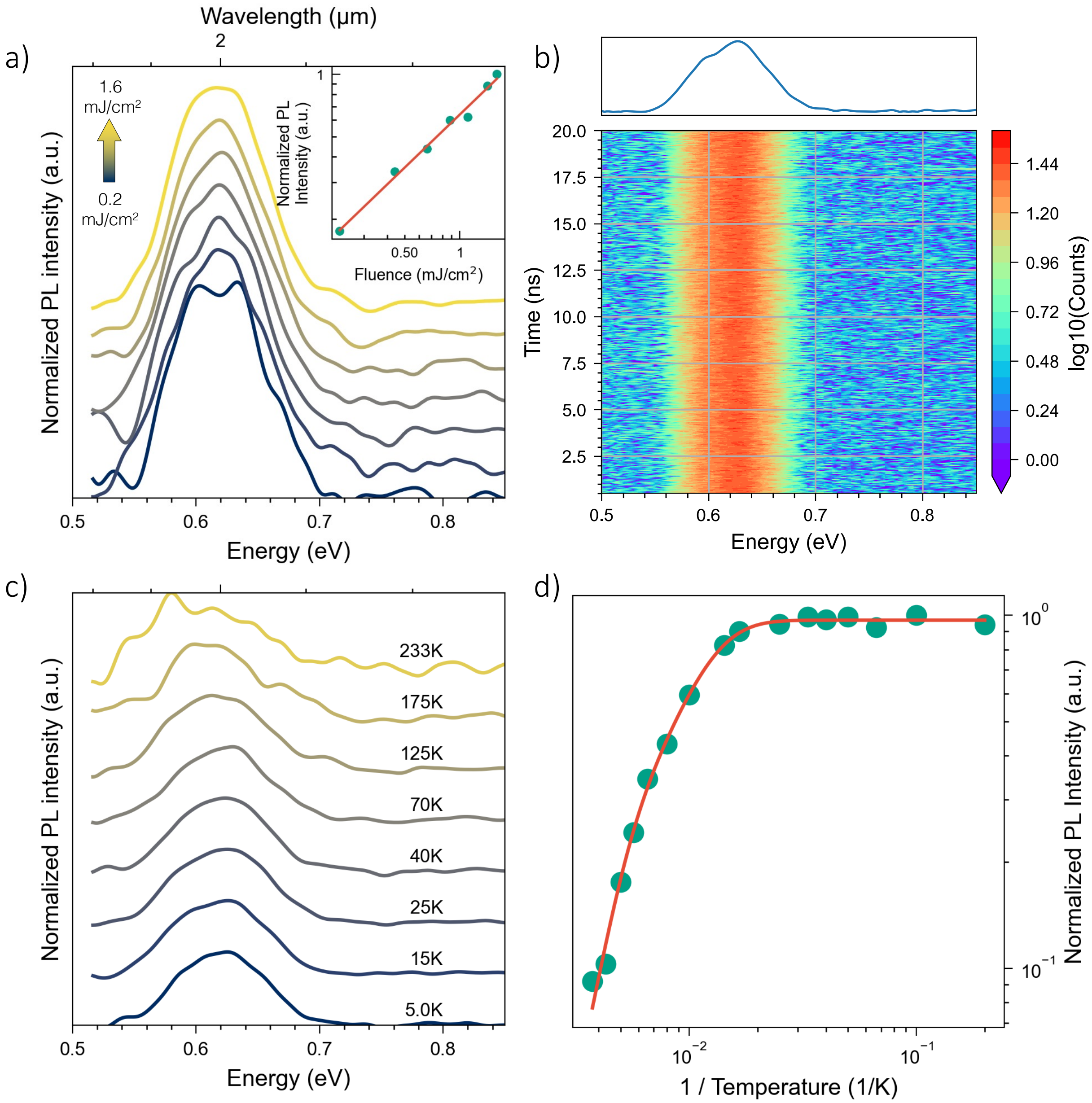

Figure S8: Optical characterization of a second as-grown 4H-Ge NWs sample. (a) Time-integrated PL spectra at 4 K using the time-resolved PL setup, as a function of excitation pulse energy (0.2–1.6 $mJ\,cm^{-2}$). The excitation densities for the individual spectra are provided in the inset and each spectrum is normalized to its own maximum. The inset shows the integrated PL intensity versus excitation density on a log–log scale (light-in–light-out curve). The red solid line is a linear fit with slope 0.85 ± 0.04. (b) Time-resolved spectral PL maps showing the time range 0.5–20 ns after excitation pulse. In this time window, a time-independent emission is observed around 0.6 eV. The marginal plot above the colored map shows the time-integrated spectrum. (c) Normalized PL spectra measured as a function of temperature. Above 230 K the emission is not detectable.(d) Integrated PL intensity versus temperature in an Arrhenius representation; the red solid line is a fit using a three-term Arrhenius model, as presented in Fig. 3c in the main text. While the emission energy and dynamics of this second sample are consistent with those presented in Fig. 3, the overall optical performance is lower. We hypothesize that this difference arises from a lower crystal quality in this sample.

PL spectra from individual NWs

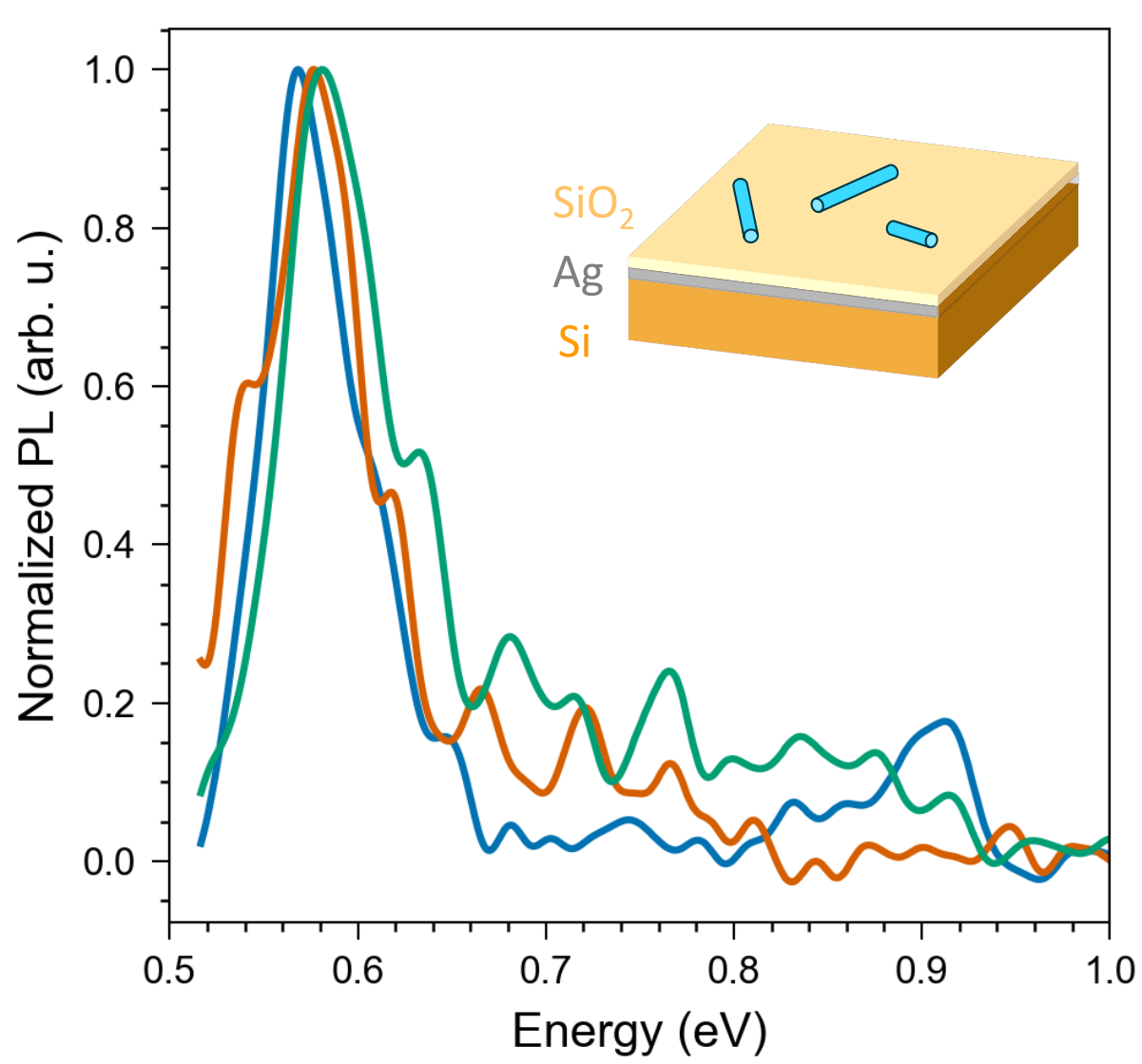

Figure S9: PL spectra from three individual 4H-Ge NWs measured with the TR-FTIR setup (see Methods). The NWs were dispersed on a reflective Si/Ag substrate coated with SiO_2 to enhance the collected PL signal. The emission peak near ~ 0.6 eV matches the ensemble PL peak in Fig. 3a in the main text, confirming the ensemble measurements. Due to the detection window cutoff at ~ 0.52 eV, part of the low-energy tail may be truncated.

Spectral lineshape analysis

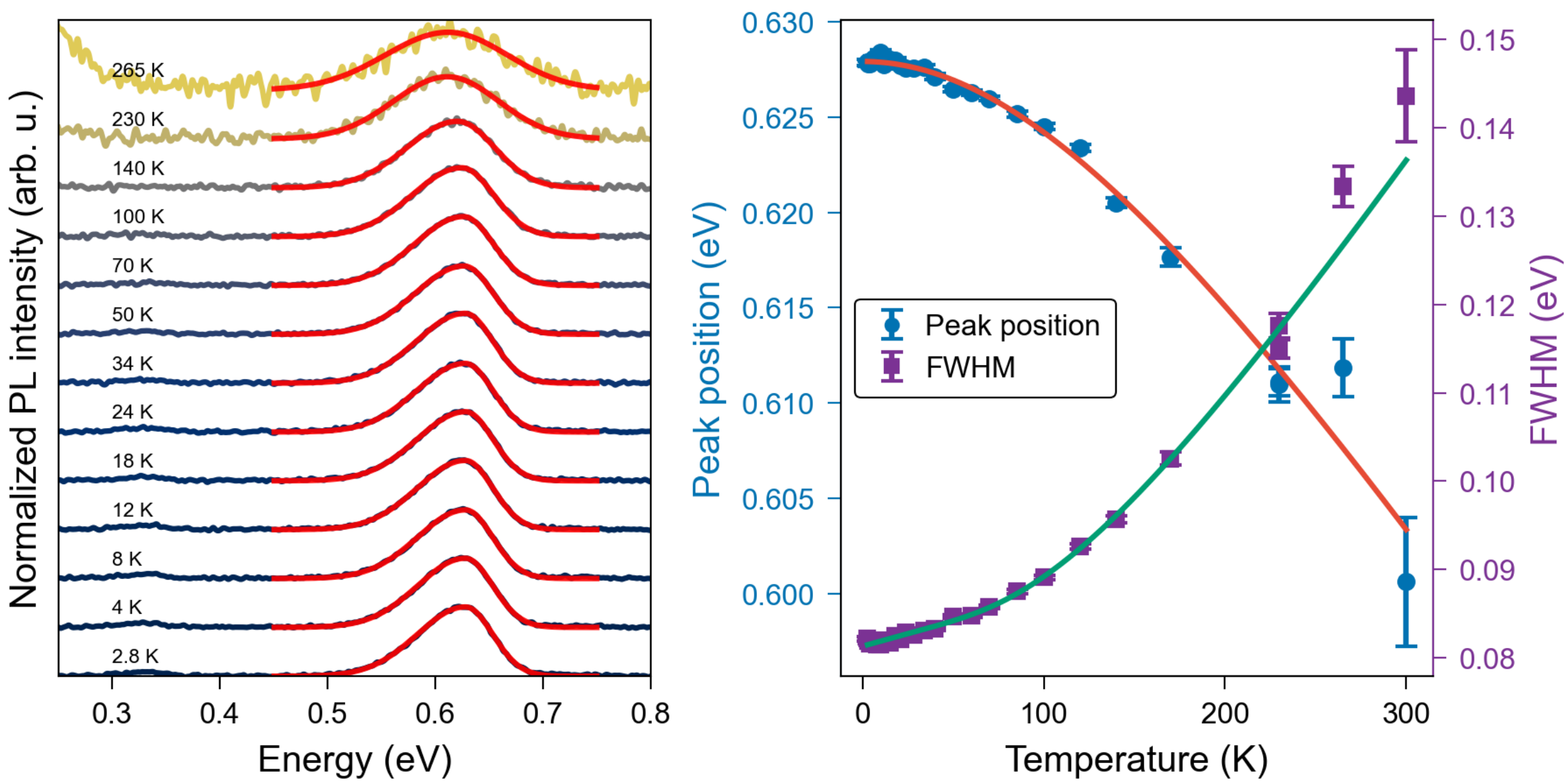

Figure S10: Fitting of the PL emission of as-grown 4H-Ge NWs depicted in Fig. 3b,d in the main text.

Here, we further analyze the monotonic decrease of the 4H-Ge bandgap with increasing temperature, as observed in Fig. 3b,d of the main text. Key parameters, such as the bandgap at 0 K, the phonon temperature and the optical phonon energy of 4H-Ge are also extracted from the evolution of the PL lineshape with temperature.

In Fig. S10a, we fit the temperature-dependent PL peak near 0.6 eV using an asymmetric Gaussian function (Eq. 1). The best-fit curve is shown as a solid red line. The fit function is given by:

$$I(E) = A \exp\left[-\frac{(E-\mu)^2}{2\,\sigma(E)^2}\right], \qquad \sigma(E) = \begin{cases} \sigma_L, & E \leq \mu, \\ \sigma_R, & E > \mu\ . \end{cases} \tag{1}$$

In Fig. S10(b), we report the peak center (μ) and the FWHM as a function of temperature, extracted from the fit parameters μ, σ_L, and σ_R (Eq. 1). The FWHM is derived as $\sqrt{2\ln 2}\,(\sigma_R + \sigma_L)$.

Phonon Temperature of 4H-Ge

The redshift of the PL peak position (μ) with temperature, shown in Fig. S10(b), can be further analyzed to extract the phonon temperature (also known as the Debye temperature) of 4H-Ge. Although this shift is commonly referred to as the Varshni shift, it is more accurately described by the semi-empirical Power-Function model developed by Pässler:[3]

$$E_g(T) = E_0 - \frac{\alpha\Theta}{2}\left[\left(1 + \left(\frac{2T}{\Theta}\right)^2\right)^{\frac{1}{2}} - 1\right] \tag{2}$$

where E_0 is the bandgap at absolute zero, α represents the limiting slope as $T \to \infty$, and Θ is the average phonon temperature. The fit is shown in Fig. S10(b) and the best parameters are reported in the upper section of Table S3.

The extracted phonon temperature of $\Theta = 400\,\mathrm{K}$ corresponds to an effective phonon energy of $k_B\Theta = (33\pm9)\,\mathrm{meV}$. Although this phonon temperature is significantly higher than the value found for 2H-Ge ($\Theta = 66\,\mathrm{K}$),[4] it is close to values typical of III–V semiconductors.[3] Furthermore, the predicted phonon energy for 4H-Ge aligns well with the 35.7 meV and 37 meV phonon energies measured in the 2H phase using Raman spectroscopy.[5]

Optical Phonon Energy of 4H-Ge

The FWHM of the bandgap related PL-emission line in Fig. S10(a) is expected to increase with temperature due to phonon scattering, as described by Rudin *et al.*:[6]

$$\Gamma(T) = \Gamma_0 + \sigma T + \frac{\Gamma_1}{\exp\left(\frac{\hbar\omega_{\mathrm{LO}}}{k_b T}\right) - 1} \tag{3}$$

where Γ_0 is the linewidth at 0 K arising from impurity and defect scattering, σ represents the acoustic phonon deformation potential interaction, $\hbar\omega_{\mathrm{LO}}$ is the longitudinal optical (LO) phonon energy, and Γ_1 is the LO-phonon coupling parameter.

The FWHM of the PL linewidth is fitted using Eq. 3. Data for the temperature-dependent

FWHM of the PL linewidth are shown in Fig. S10b, together with the fitting curve. The resulting fitting parameters are presented in the lower section of Table S3. The extracted LO-phonon energy is $\hbar\omega_{\mathrm{LO}} = (33 \pm 3)\,\mathrm{meV}$, which is in excellent agreement with the LO-phonon energy deduced from the fit of the temperature dependence of the bandgap.

Finally, our extracted phonon energy for 4H-Ge is consistent with the 30 meV to 38 meV range predicted by theory.[7,8]

Table S3: Extracted parameters from fitting the temperature-dependent bandgap shift (upper section) and the PL linewidth variation (lower section).

Parameter	Value
E_0	$(629.92 \pm 0.07)\,\mathrm{meV}$
α	$(0.15 \pm 0.03)\,\mathrm{meV\,K^{-1}}$
Θ	$(4 \pm 1) \times 10^2\,\mathrm{K}$
Γ_0	$(81.3 \pm 0.1)\,\mathrm{meV}$
σ	$(56 \pm 5)\,\mathrm{\mu eV\,K^{-1}}$
Γ_1	$(0.10 \pm 0.02)\,\mathrm{eV}$
$\hbar\omega_{\mathrm{LO}}$	$(33 \pm 3)\,\mathrm{meV}$

PL from 4H-SiGe

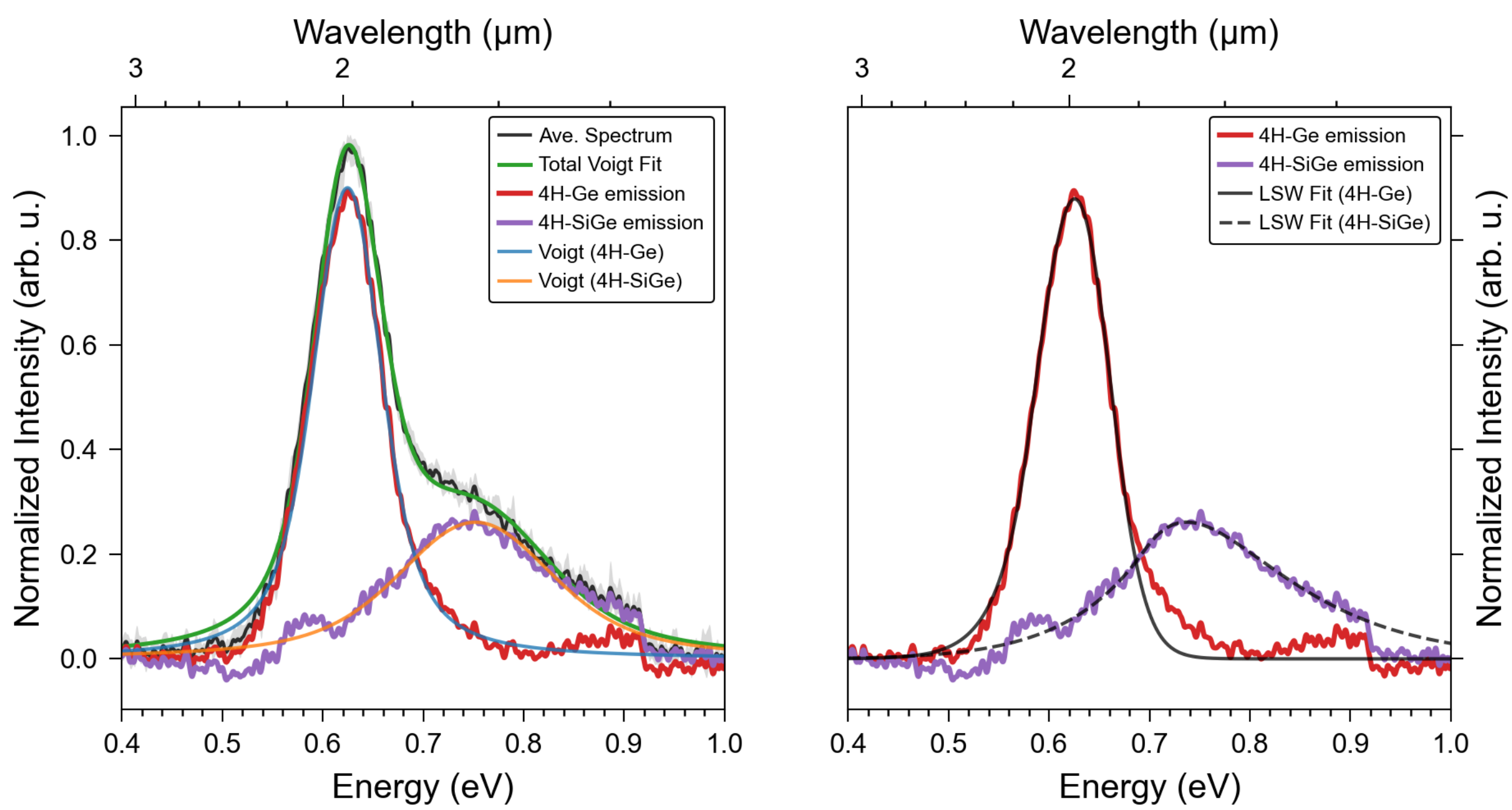

Figure S11: Fit of the PL of the 4H-SiGe sample. (a) The average experimental spectrum (solid black line; the shaded gray area indicates the error of the average) is fitted using a double Voigt profile (green line). The individual Voigt components for 4H-SiGe and 4H-Ge are shown in red and purple, respectively. (b) The isolated spectra extracted for 4H-Ge and 4H-SiGe are plotted in red and purple. The black solid and dashed lines are the Lasher–Stern–Würfel (LSW) fits.

As depicted in Fig. 2b, the 4H-$Si_{0.2}Ge_{0.8}$ NWs are grown on an initial 4H-Ge segment. Consequently, the measured spectrum Fig. 4a exhibits two distinct emission peaks: one originating from the 4H-Ge segment (which matches the behavior shown in Fig. 3a and a higher-energy peak from the higher bandgap 4H-SiGe segment. To analyze the 4H-SiGe emission independently, we fit the total spectrum using a double Voigt profile (Fig. S11). The isolated spectra for each semiconductor were extracted by subtracting the fitted Voigt component of the other semiconductor from the total signal. To obtain the bandgap of our material, we fit a Lasher–Stern–Würfel model as explained in Fig. S7 to both the 4H-Ge and SiGe individual spectra, as shown in Fig. S11b. The estimated bandgaps are 0.577 ± 0.001 eV and 0.691 ± 0.001 eV for 4H-Ge and 4H-$Si_{0.2}Ge_{0.8}$, respectively.

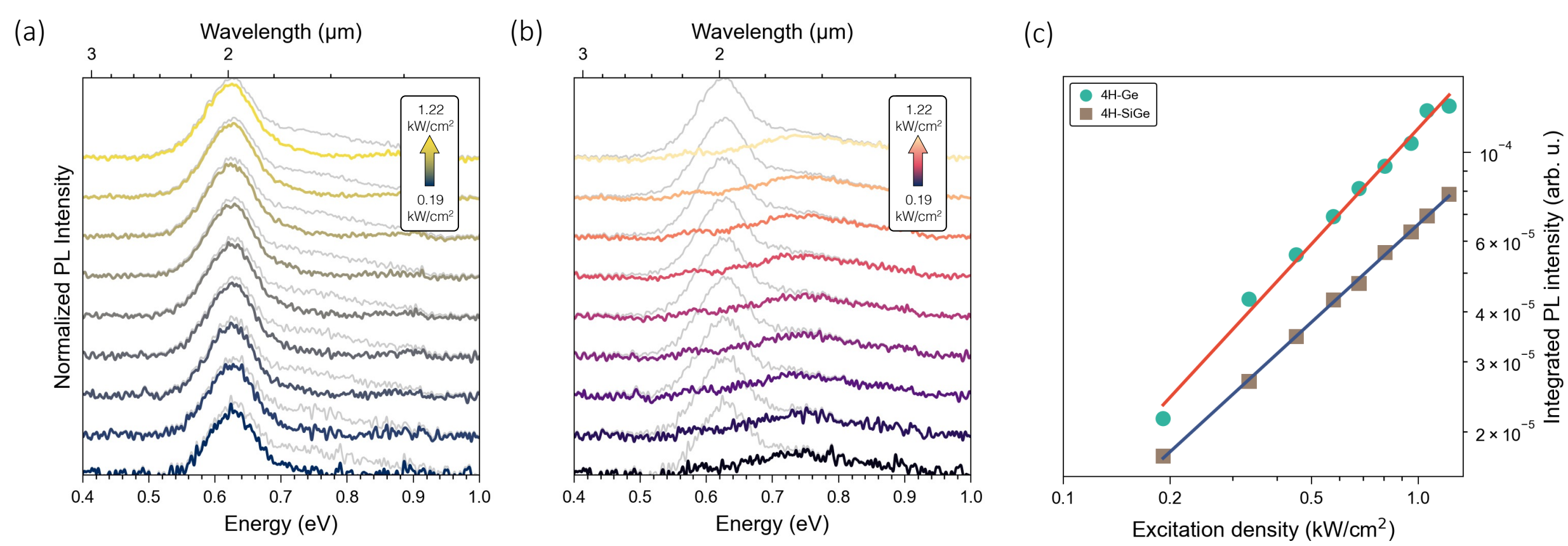

Figure S12: Isolated power dependent PL spectra for 4H-Ge and 4H-SiGe. Excitation-power dependence of the PL spectra for (a) 4H-Ge and (b) 4H-SiGe. (c) Light-in-light-out plot showing a slope of 0.97 ± 0.03 for 4H-Ge (green circles) and 0.812 ± 0.008 for 4H-SiGe (grey squares).

Figs. S12a and S12b show the excitation power-dependent PL for 4H-Ge and 4H-SiGe. The corresponding light-in-light-out plot (Fig. S12c) yields a slope of 0.97 ± 0.03 for 4H-Ge,

consistent with the analysis in the inset of Fig. 3a. The 4H-SiGe slope is noticeably lower at 0.812 ± 0.008. We attribute this reduction to the sample geometry and long carrier lifetimes, which may allow carriers generated in the 4H-SiGe region to diffuse into the lower-bandgap 4H-Ge segment before recombining.

Time Resolved PL of 4H-Ge

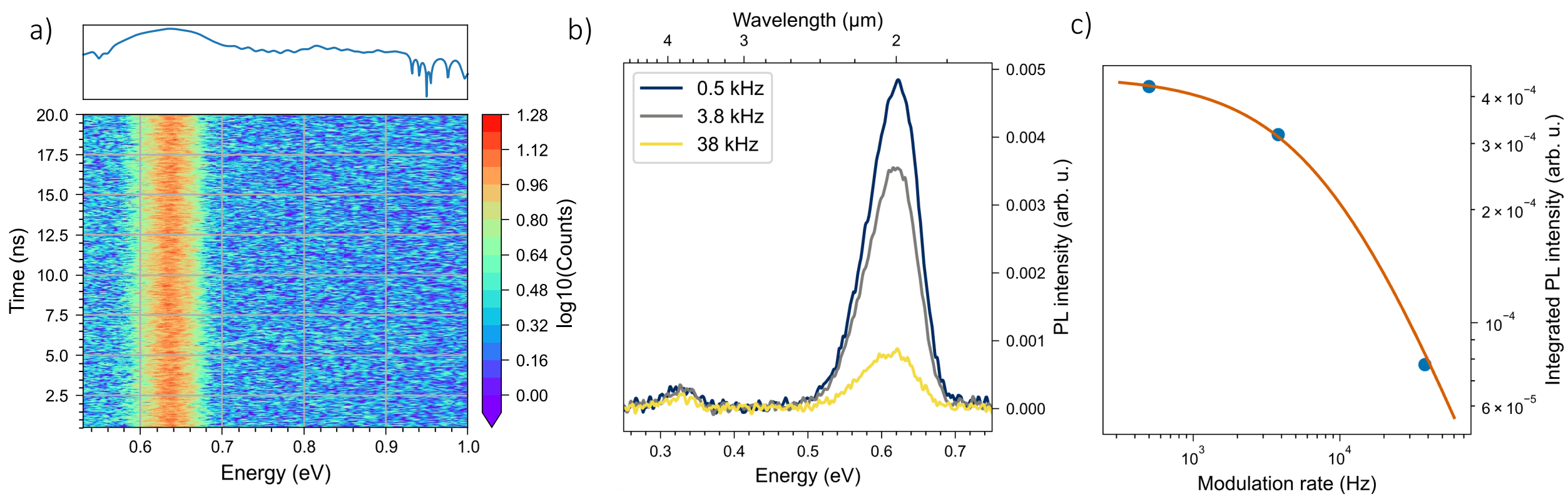

Figure S13: Lifetime measurements on as-grown 4H-Ge NWs. (a) Time-resolved spectral PL maps showing the time range 0.5–20 ns after excitation pulse, where a nearly constant emission is observed around 0.6 eV. The marginal plot above the colored map shows the time-integrated spectrum on a logarithmic scale. (b) PL spectra acquired at three excitation modulation frequencies. (c) Integrated intensity of the 4H-Ge peak as a function of modulation frequency; the red solid line is a fit to a first-order low-pass response (Eq. 1)

Fig. 4c of the main text highlights the dependence of the PL intensity on the modulation frequency for 4H-Ge and 4H-SiGe, implying very long lifetimes on the order of tens to hundreds of µs for 4H-Ge and 4H-$Si_{0.2}Ge_{0.8}$, respectively. To further investigate the behavior of these samples and confirm the reproducibility of the observed effect, we conducted time-resolved (TR) PL measurements on a 4H-Ge sample shown in Fig. 3. The technique is described in the Methods section. Fig. S13a shows a typical time-resolved PL map, where no decay of the PL is observable during the first 20 ns after the excitation pulse, indicating a lifetime $\gg$ 20 ns.

Figs. S13b and c present the dependence of the PL intensity on the modulation rate of the excitation laser. Similarly to the observations in Fig. 4c of the main text, the PL rapidly

quenches at higher modulation frequencies. By fitting the integrated PL as a function of the modulation rate with Eq. 1 in the Methods section of the main text, we obtain a lifetime of 120 ± 10 µs. As discussed in the main text, this observation is in agreement with theory[9] and is significantly longer than the reported lifetimes of ≈ 1 ns in 2H-Ge and 2H-SiGe.[4,10]

Initial PL Decay

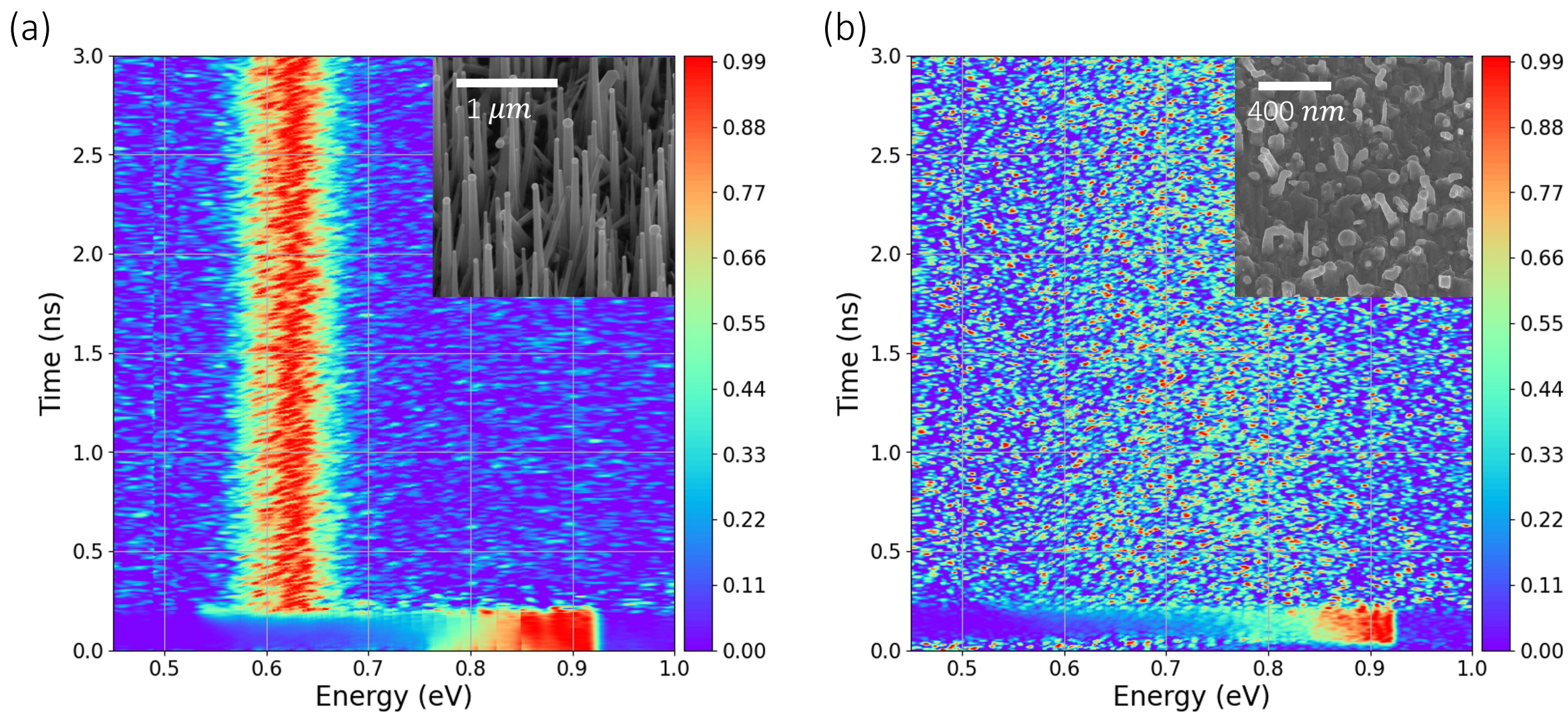

Figure S14: Time-resolved spectral PL maps showing the time range 0–3 ns after the excitation pulse for (a) a successful 4H-Ge NW sample and (b) a sample with many inclined NWs, where no 4H-Ge NWs were found. The graph is normalized to unity along the energy axis. The inset in each panel shows a Scanning Electron Microscopy image of the sample morphology.

The initial part of the PL decay immediately following the excitation laser pulse exhibits a broad emission. This emission quickly quenches, giving way to a time-independent emission located at the respective bandgap energies of 4H-Ge and 4H-SiGe. In this section, we provide insight into this initial PL emission and hypothesize about its origin.

In Fig. S14(a), we report the time-resolved spectral PL map for a 4H-Ge sample focusing on the initial 3 ns after the excitation pulse. Here, we observe a time-independent PL emission around 0.62 eV, attributed to the 4H-Ge NWs, alongside a broad PL transient of unknown origin and limited by the filter cutoff at 0.92 eV , which decays within 250 ps.

To determine the origin of this transient emission, we repeated the analysis on a sample with many inclined NWs with a cubic crystal structure. A representative SEM image of the sample morphology is shown in the inset of panel (b). A very similar initial PL transient is also observed in this sample. However, this sample only contain cubic NWs, no time-independent emission from the 4H phase is detected around 0.62 eV. This indicates that the initial PL transient does not originate from the 4H-Ge NWs but rather from the the cubic NWs or the substrate, which fall outside the scope of this paper.

Ab initio calculations

The modeling of a binary alloy A_xB_{1-x}, was performed within the strict-regular solution (SRS) model,[11,12] under which an alloy is described as a combination of non-equivalent ordered unit cells. Within this approximation, a given property of the macroscopic alloy ($\langle P\rangle$) is written as an average of the properties of the ordered unit cells (P_i),

$$\langle P\rangle = \sum_i x_i^0 P_i \tag{4}$$

with the weights of each unit cell (x_i^0) given by

$$x_i^0 = g_i x^{n_\mathrm{A}}(1-x)^{n-n_\mathrm{A}} \tag{5}$$

where n (n_A) is the number of atoms (of species A) in the unit-cell, x is the macroscopic concentration of species A in the alloy and g_i is the degeneracy of the unit-cell (i.e. the number of symmetry equivalent structures). For this purpose, we use the 76 unit cells corresponding to the non-equivalent atomic permutations of the primitive 4H cell.

All *ab initio* calculations were performed within the projector-augmented wave formalism[13] using the Vienna Ab-initio Simulation Package[14] (VASP; version 6.4.2), using the POTPAW set version 6.4.[15] For Si the last s and p shells are set in valence ($3s^23p^2$), while

for Ge the d-states are also included (i.e. $3d^{10}4s^24p^2$). A cutoff of 520 eV was used throughout the work.

Geometry optimizations were performed using the Perdew-Burke-Ernzenhof approximation revised for solids (PBEsol),[16] until all forces acting on atoms were smaller than 1 meV Å^{-1} and stresses smaller than 0.1 kbar using Γ-centered Monkhorst-Pack grids[17] generated with a spacing of 0.09 Å where used. These calculations were performed within the collinear-spin regime, starting from the high-spin ferromagnetic configuration.

For the calculations of electronic structure we used the modified Becke-Johnson approximation (mBJ)[18] to the exchange-correlation potential. In addition, due to the importance of spin-orbit coupling to the correct description of the spin-split valence band (and the associated transition) this effect was also included.

Radiative lifetimes (τ) for individual unit cells were computed based on a thermal averaging of the radiative recombination rate $A_{cv\mathbf{k}}$ for vertical optical transitions between a conduction state $|c\mathbf{k}\rangle$ and a valence state $|v\mathbf{k}\rangle$ with the one-particle energies $\varepsilon_{c\mathbf{k}}$ and $\varepsilon_{v\mathbf{k}}$. Assuming the emission of unpolarized light (i.e. averaging over the Cartesian directions), $A_{cv\mathbf{k}}$ is written as[19,20]

$$A_{cv\mathbf{k}} = n_{\text{eff}} \frac{e^2(\varepsilon_{c\mathbf{k}} - \varepsilon_{v\mathbf{k}})}{\pi\epsilon_0\hbar^2 m^2 c^3} \frac{1}{3} \sum_{j=1}^{3} |\langle c\mathbf{k}|p_j|v\mathbf{k}\rangle|^2 \,, \tag{6}$$

where n_{eff} is the effective refractive index of the medium. The decay rate $1/\tau$ can then be approximated as the thermal average of the recombination rates at temperature T,

$$\frac{1}{\tau} = \sum_{cv\mathbf{k}} A_{cv\mathbf{k}} \frac{w_{\mathbf{k}}\, e^{-(\varepsilon_{c\mathbf{k}}-\varepsilon_{v\mathbf{k}})/(k_{\text{B}}T)}}{\sum\limits_{c'v'\mathbf{k}'} w_{\mathbf{k}'}\, e^{-(\varepsilon_{c'\mathbf{k}'}-\varepsilon_{v'\mathbf{k}'})/(k_{\text{B}}T)}} \tag{7}$$

with $\mathbf{k}$-point weights $w_{\mathbf{k}}$. Since the refractive index does not vary significantly across frequencies, we took the average of the static refractive index of the corresponding material as a reference for n_{eff}. As noted in previous works,[21] Eq. (7) may require extremely dense k-

point grids to ensure the correct convergence of τ, as is the case when $A_{cv\mathbf{k}}$ depends strongly on $\mathbf{k}$ (e.g. the largest elements are located around specific regions of reciprocal space). In such situations uniform meshes are inefficient in sampling the reciprocal space, hence the high-density required. To bypass this one could use non-uniform grids,[22] but since Eq. (7) does not involve transitions at different k-points it is cheaper to perform several calculations at uniform but disjoint grids and merge them afterwards. The weight of each k-point in this non-uniform grid is simply given by the volume of the corresponding cell in the Voronoi tessellation of the reciprocal space,[23] normalized to the volume of the Brillouin Zone. As such we performed four separate calculations for each alloy using Γ-centered and $(0, 0, 1/2)$-shifted Mohnkhost-Pack grids with sizes $24 \times 24 \times 6$ and $19 \times 19 \times 11$. These grids are almost co-prime (the Γ point will be shared by the centered grids) and allow a good sampling of both the basal plane and the more important Γ-A region of the Brillouin zone.

The radiative lifetime of the macroscopic alloy at concentration x was computed in the style of Mathiessen's rule, by averaging the effective recombination rate of each individual unit cell (using the weights from the SRS),

$$\left\langle \frac{1}{\tau} \right\rangle_{\mathrm{SRS}} = \sum_{i=1}^{N_{\mathrm{cell}}} \frac{1}{\tau_i} x_i^0 \,. \tag{8}$$

with x_i^0 given by Eq. (5), and inverting the resulting value, i.e. $\langle \tau \rangle = \langle 1/\tau \rangle_{\mathrm{SRS}}^{-1}$.

References

(1) Li, A.; Hauge, H. I.; Verheijen, M. A.; Bakkers, E. P.; Tucker, R. T.; Vincent, L.; Renard, C. Hexagonal silicon-germanium nanowire branches with tunable composition. *Nanotechnology* **2023**, *34*, 015601.

(2) Vincent, L.; Fadaly, E. M.; Renard, C.; Peeters, W. H.; Vettori, M.; Panciera, F.; Bouchier, D.; Bakkers, E. P.; Verheijen, M. A. Growth-Related Formation Mechanism

of I_3-Type Basal Stacking Fault in Epitaxially Grown Hexagonal Ge-2H. *Advanced Materials Interfaces* **2022**, *9*, 2102340.

(3) Pässler, R. Semi-empirical descriptions of temperature dependences of band gaps in semiconductors. *Physica Status Solidi (B) Basic Research* **2003**, *236*, 710–728.

(4) Fadaly, E. M. et al. Direct-bandgap emission from hexagonal Ge and SiGe alloys. *Nature* **2020**, *580*, 205–209.

(5) De Matteis, D.; De Luca, M.; Fadaly, E. M.; Verheijen, M. A.; López-Suárez, M.; Rurali, R.; Bakkers, E. P.; Zardo, I. Probing Lattice Dynamics and Electronic Resonances in Hexagonal Ge and Si_xGe_{1-x} Alloys in Nanowires by Raman Spectroscopy. *ACS Nano* **2020**, *14*, 6845–6856.

(6) Rudin, S.; Reinecke, T. L. Temperature-dependent exciton linewidths in semiconductor quantum wells. *Physical Review B* **1990**, *41*, 3017–3027.

(7) Kiefer, F.; Hlukhyy, V.; Karttunen, A. J.; Fässler, T. F.; Gold, C.; Scheidt, E. W.; Scherer, W.; Nylén, J.; Häussermann, U. Synthesis, structure, and electronic properties of 4H-germanium. *Journal of Materials Chemistry* **2010**, *20*, 1780–1786.

(8) López-Cruz, E.; Cardona, M. Raman spectra of two new modifications of germanium: Allo-germanium and 4H-Ge. *Solid State Communications* **1983**, *45*, 787–789.

(9) Keller, M.; Belabbes, A.; Furthmüller, J.; Bechstedt, F.; Botti, S. First-principles insight in structure-property relationships of hexagonal Si and Ge polytypes. *Physical Review Materials* **2023**, *7*, 064601.

(10) van Lange, V. T.; Dijkstra, A.; Fadaly, E. M.; Peeters, W. H.; van Tilburg, M. A.; Bakkers, E. P.; Bechstedt, F.; Finley, J. J.; Haverkort, J. E. Nanosecond Carrier Lifetime of Hexagonal Ge. *ACS Photonics* **2024**, *11*, 4258–4267.

(11) Chen, A.-B.; Sher, A. *Semiconductor Alloys: Physics and Materials Engineering*; Microdevices; Plenum Press: New York, 1995.

(12) Sher, A.; van Schilfgaarde, M.; Chen, A.-B.; Chen, W. Quasichemical Approximation in Binary Alloys. *Phys. Rev. B* **1987**, *36*, 4279–4295.

(13) Kresse, G.; Joubert, D. From Ultrasoft Pseudopotentials to the Projector Augmented-Wave Method. *Phys. Rev. B* **1999**, *59*, 1758–1775.

(14) Kresse, G.; Furthmüller, J. Efficient Iterative Schemes for *Ab Initio* Total-Energy Calculations Using a Plane-Wave Basis Set. *Phys. Rev. B* **1996**, *54*, 11169–11186.

(15) The SHA256 id of the POTCARs are for Si 79d9987ad8750f624c4d6acb2a16d13abf6a777132adc04dc6c8399be72b42bb and for Ge 944b26c40d2d7c4f4eb1ff3a2e8dbfdd7129146276cd341854bfd5c0e57780ed.

(16) Perdew, J. P.; Ruzsinszky, A.; Csonka, G. I.; Vydrov, O. A.; Scuseria, G. E.; Constantin, L. A.; Zhou, X.; Burke, K. Restoring the Density-Gradient Expansion for Exchange in Solids and Surfaces. *Phys. Rev. Lett.* **2008**, *100*, 136406.

(17) Monkhorst, H. J.; Pack, J. D. Special points for Brillouin-zone integrations. *Physical review B* **1976**, *13*, 5188.

(18) Tran, F.; Blaha, P. Accurate Band Gaps of Semiconductors and Insulators with a Semilocal Exchange-Correlation Potential. *Phys. Rev. Lett.* **2009**, *102*, 226401.

(19) Delerue, C.; Allan, G.; Lannoo, M. Theoretical Aspects of the Luminescence of Porous Silicon. *Phys. Rev. B* **1993**, *48*, 11024–11036.

(20) Dexter, D. In *Solid State Physics*; Seitz, F., Turnbull, D., Eds.; Academic Press, 1958; Vol. 6; p 353.

(21) Rödl, C.; Furthmüller, J.; Suckert, J. R.; Armuzza, V.; Bechstedt, F.; Botti, S. Accurate electronic and optical properties of hexagonal germanium for optoelectronic applications. *Physical Review Materials* **2019**, *3*, 034602.

(22) da Jornada, F. H.; Qiu, D. Y.; Louie, S. G. Nonuniform sampling schemes of the Brillouin zone for many-electron perturbation-theory calculations in reduced dimensionality. *Physical Review B* **2017**, *95*, 035109.

(23) Chen, S.; Salzbrenner, P. T.; Monserrat, B. Nonuniform grids for Brillouin zone integration and interpolation. *Physical Review B* **2022**, *106*, 155102.
